# The effect of chronic stress and chronic alcohol intake on behaviour, brain volume, and functional connectivity in a longitudinal rat model

**DOI:** 10.1093/braincomms/fcaf432

**Published:** 2025-11-01

**Authors:** Jalil Rasgado-Toledo, Diego Angeles-Valdez, César J Carranza-Aguilar, Alejandra Lopez-Castro, Luis A Trujillo-Villarreal, David Medina-Sánchez, Mariana S Serrano-Ramirez, A Débora Elizarrarás-Herrera, Sarael Alcauter, Ilse Delint-Ramirez, Ranier Gutierrez, Gabriel A Devenyi, M Mallar Chakravarty, Eduardo A Garza-Villarreal

**Affiliations:** Institute of Neurobiology, Universidad Nacional Autónoma de México campus Juriquilla, Santiago de Querétaro, Querétaro 76230, Mexico; Institute of Neurobiology, Universidad Nacional Autónoma de México campus Juriquilla, Santiago de Querétaro, Querétaro 76230, Mexico; Center for Clinical Neuroscience and Cognition, University Medical Center Groningen, University of Groningen, Groningen 9713 GZ, The Netherlands; Research School of Behavioural and Cognitive Neurosciences, University of Groningen, Groningen 9713 AV, The Netherlands; Institute of Neurobiology, Universidad Nacional Autónoma de México campus Juriquilla, Santiago de Querétaro, Querétaro 76230, Mexico; Institute of Neurobiology, Universidad Nacional Autónoma de México campus Juriquilla, Santiago de Querétaro, Querétaro 76230, Mexico; Institute of Neurobiology, Universidad Nacional Autónoma de México campus Juriquilla, Santiago de Querétaro, Querétaro 76230, Mexico; Institute of Neurobiology, Universidad Nacional Autónoma de México campus Juriquilla, Santiago de Querétaro, Querétaro 76230, Mexico; Institute of Neurobiology, Universidad Nacional Autónoma de México campus Juriquilla, Santiago de Querétaro, Querétaro 76230, Mexico; Institute of Neurobiology, Universidad Nacional Autónoma de México campus Juriquilla, Santiago de Querétaro, Querétaro 76230, Mexico; Institute of Neurobiology, Universidad Nacional Autónoma de México campus Juriquilla, Santiago de Querétaro, Querétaro 76230, Mexico; Departments of Psychiatry, University of Texas Southwestern Medical Center, Dallas, TX 75390, USA; Departments of Neuroscience, University of Texas Southwestern Medical Center, Dallas, TX 75390, USA; Departments of Cell Biology, University of Texas Southwestern Medical Center, Dallas, TX 75390, USA; Department of Pharmacology, Laboratory of Neurobiology of Appetite, Center for Research and Advanced Studies of the National Polytechnic Institute, Mexico City 07360, Mexico; Center for Aging Research CIE, Cinvestav Sede Sur, Mexico City 07360, Mexico; Department of Psychiatry, McGill University, Montreal, QC, Canada H3A 1A1; Cerebral Imaging Centre, Douglas Mental Health University Institute, Montreal, QC, Canada H4H 1R3; Department of Psychiatry, McGill University, Montreal, QC, Canada H3A 1A1; Cerebral Imaging Centre, Douglas Mental Health University Institute, Montreal, QC, Canada H4H 1R3; Institute of Neurobiology, Universidad Nacional Autónoma de México campus Juriquilla, Santiago de Querétaro, Querétaro 76230, Mexico

**Keywords:** chronic restraint stress (CRS), alcohol use disorder (AUD), IA2BC, animal models, longitudinal MRI

## Abstract

Pathological chronic stress is stress exceeding the organism's ability to cope physiologically, which may act as a risk factor in the onset and relapse of alcohol use disorder. Chronic-restraint stress (CRS) and ethanol intake are independently known to induce changes in brain structure and function, however, their combined effects on neurodevelopment over long periods of time remains largely unexplored. We conducted an in vivo longitudinal rat model with three main goals. 1) to determine if chronic stress increases ethanol intake; 2) to determine the effect of chronic- stress and ethanol intake in behavioural measures, brain structure, and function; and 3) to investigate the effect of sex. This observational study included Wistar rats assigned to four groups: 1) ethanol consumption (EtOH+/CRS−), 2) stress exposure (EtOH−/CRS+), 3) both ethanol and stress exposure (EtOH+/CRS+), and 4) control group (EtOH−/CRS−). Our results showed that chronic stress did not affect ethanol intake but led to reduced body weight gain, elevated corticosterone levels, and impaired recognition memory. Structural MRI revealed that both exposures produced additive brain volume changes in olfactory bulb, orbitofrontal cortex, caudate-putamen, hippocampus, and cerebellum. Functional connectivity analysis using network-based statistics identified disrupted cortical-subcortical connections. Results found here were sex-dependent in terms of volumetric changes (higher effects on males) and functional connectivity (higher effects on females). Findings suggest sex-dependent mechanisms where both chronic- ethanol intake and stress affect brain plasticity during neurodevelopment. Understanding region-specific vulnerabilities is crucial for addressing alcohol use disorders and stress-related neuropathology.

## Teaser

Rasgado-Toledo, *et al*. reported sex-dependent and region-specific effects of combined ethanol exposure and chronic stress on both the rat brain volume and functional networks, affecting its plasticity during neurodevelopment.

## Graphical Abstract

**Graphical Abstract:**
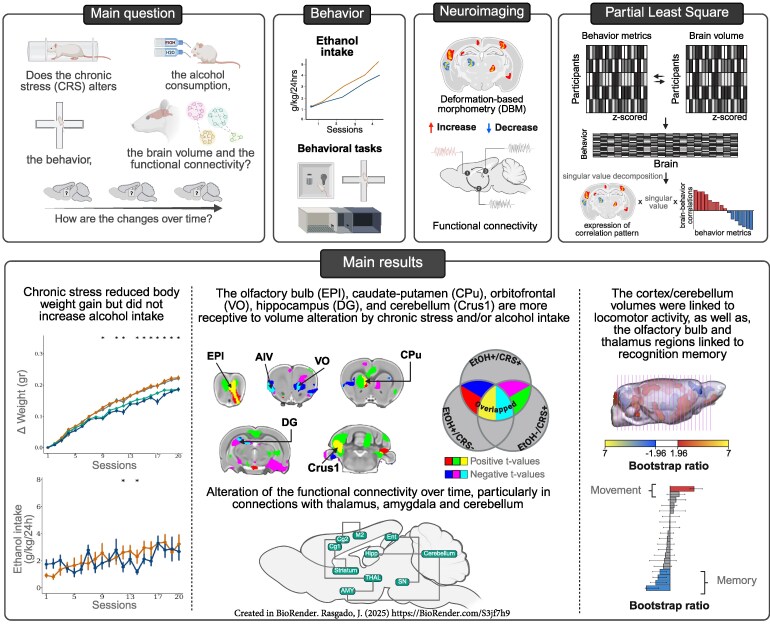

## Introduction

Stress can be defined as the physical or psychological result of an organism's exposure to adverse experiences and internal or external demands. Based on the chronology, it can also be defined as acute or chronic, the latter of which can become pathological.^1^ Pathological chronic stress is described as stress exceeding the organism's ability to cope physiologically, which may act as a risk factor in the onset and relapse of several neurological and psychiatric disorders, including substance use disorders (SUDs).^2-4^ The chronicity of stressful events lead to a disruption of the finely regulated hypothalamic-pituitary-adrenal (HPA) axis and norepinephrine/autonomic systems such as locus coeruleus-noradrenergic (LC-NA) network,^5^ leading to neurochemical alterations associated with stress responses.^6,7^ This disruption eventually entails an increase in behaviour changes, such as alterations in anxiety levels, negative emotions, altered sleep patterns and appetite fluctuations, aggressive behaviours, and shifts in attention, concentration, and memory.^8^ These disruptions often can lead to substance use and abuse as a way to cope with these negative emotional states.^9^

Alcohol use disorder (AUD) has been largely associated with acute and chronic stress, with the prevalence of stress-induced alcohol use and its role as a coping mechanism being well-documented in human studies.^2,4^ Several rodent studies have demonstrated that exposure to acute and sub-chronic stress increased alcohol (ethanol) intake.^10^ However, contradictory findings have been reported in other studies, which demonstrated that, acute and sub-chronic stress exposure led to a reduction in ethanol intake.^10^ Furthermore, only a limit studies have investigated the effects of chronic stress, mainly finding it has no effect on ethanol intake.^10^ The possible mechanisms underlying stress and ethanol intake are not well defined and seem to be complex. Whether stress induces changes in ethanol intake seems to be dependent on the type of stressor, its intermittency, predictability, chronicity, and biological variables such as genetics, age, and sex.^2,10,11^ Furthermore, recent studies have been posited that individual differences in anxiety traits and coping mechanisms for negative internal states derived from stress may serve as a potential moderating factor in the increased vulnerability to the development of compulsive alcohol consumption.^12-14^ Moreover, different studies have documented the presence of subgroups exhibiting disparate drinking patterns.^12,15,16^ These patterns are evident not solely in quantity measures but also in behavioural traits such as impulsivity, decision-making processes, and Pavlovian-conditioning.^17,18^ Despite this complexity, the underlying and shared neural circuits may offer a unifying explanation for the relationship between stress and ethanol intake.^8^ This hypothesis has been postulated based on neuroimaging studies in humans showing that changes in volume and function of specific brain regions can predict the development and relapse of AUD.^8,19-22^

Neuroimaging studies in rodent models of alcohol abuse have shown that chronic ethanol intake reduced the volume of specific brain regions such as hippocampus, thalamus, ventral tegmental area, substantia nigra, caudate-putamen, nucleus accumbens, retrosplenial, somatosensory, insular and prelimbic cortex, and increased the volume of orbitofrontal cortex, thalamus and cerebellum.^23-26^ Longitudinal studies have shown that continuous ethanol intake can result in alterations to the volume of these regions, with a either shrinkage or an enlargement pattern in comparison to the typical expected change.^27,28^ Functional neuroimaging (fMRI) studies revealed that chronic ethanol intake also induces a decrease in connectivity patterns between prefrontal cortical subregions, frontostriatal connectivity, and retrosplenial-visual networks, whereas between the prefrontal to cingulate and striatal networks increases.^29,30^ Conversely, cross-sectional and longitudinal chronic stress studies have described only a reduced in volume within the hippocampus, prelimbic, cingulate, insular, somatosensory, motor, auditory, and perirhinal/entorhinal cortices, as well as the striatum, nucleus accumbens, the bed nucleus of the stria terminalis, amygdala, and the thalamus.^31-34^ fMRI studies have shown increased connectivity within functional networks such as the somatosensory, visual, and default mode,^35^ and increased connectivity of thalamic connections to the hippocampus, amygdala, ventral tegmental area, prelimbic, insular and retrosplenial cortices.^31^ Consequently, individual studies have identified brain regions that appear to be affected in both pathologies. However, there is a paucity of studies that simultaneously investigate both and the potential synergies between them.

Sex-differences on the long-term influence of stress on ethanol intake have been described but are not well understood. For the most part, human research has described different coping mechanisms for SUD-related drinking behaviour, possibly due to sexual differences in terms of neurochemical transmission, genetics, brain morphology, and connectivity.^36^ New insights derived from different species addressing the ethanol influence in the context of intoxication, withdrawal, and cravings showed age and sex may act as mediators in different directions by either increasing or decreasing vulnerability to AUD and may serve as predictors of the negative impact on the brain.^37^ Moreover, chronic stress studies have emphasized that the sex-specific impacts are also dependent on the drinking onset age, possibly driven by gonadal hormone fluctuations and the subsequent HPA/LC-NA responsiveness that occur over dynamic periods of development and maturation.^38^

In light of these considerations, the objectives of our study were as follows: first, to ascertain whether chronic stress exacerbates ethanol intake by employing a pre-clinical model of alcohol abuse and dependence; second, to determine the impact of chronic stress and chronic ethanol intake on behavioural measures, brain structure and connectivity; and third, to investigate the effect of sex. To this end, a longitudinal design and in vivo magnetic resonance imaging were employed in rat models of chronic stress and chronic ethanol intake.

## Materials and methods

### Animals

**Figure 1:**
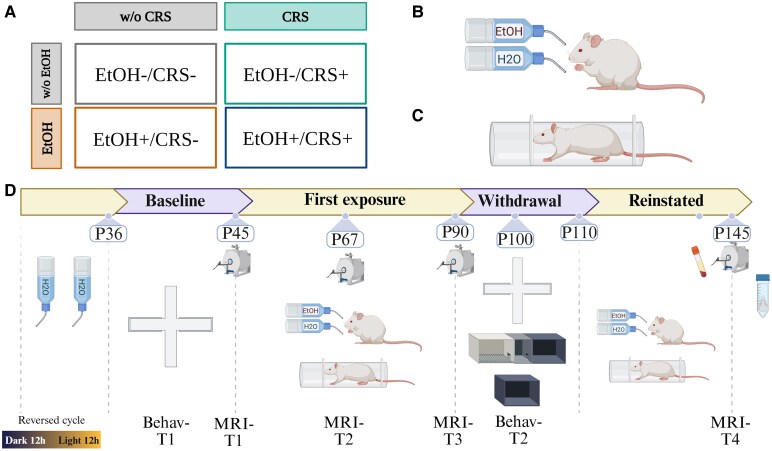
**Experimental design.** (**A**) Representative group subdivision. (**B**) Schematic drawing illustrating the intermittent access model. (**C**) Schematic drawing of movement restrictor with adjustment for rat size. (**D**) Experimental protocol: P21, rats were received; P35, baseline behavioural evaluation (Behav-T1); P44, first MRI acquisition (MRI-T1); P45, initiation of IA2BC model during 45 days; P67, second MRI acquisition (MRI-T2); P91, third MRI acquisition (MRI-T3); P100, second behavioural evaluation (Behav-T2: EPM, CPP and NOR in this order); P91 to P110, withdrawal phase; P110, reinstaining of both interventions; P145, four MRI acquisition (MRI-T4). Abbreviations: EtOH, ethanol intake; CRS, chronic restraint stress; H2O, water intake; MRI, magnetic resonance imaging. Created in BioRender. Rasgado, J. (2025) https://BioRender.com/1f7152h.

A total of 96 Wistar rats (45 females) included in the experiment were received at postnatal day twenty-one (P21). The study was conducted over six batches of animals, with approximately 16 rats per batch. The rats were randomly assigned to four experimental groups. This was achieved by extracting a random Rat-ID from the total number of rats in the batch, without replacement. Group 1 ethanol consumption (*EtOH+/CRS−*, *n* = 28, 13 females) consisted of animals under the Intermittent Access 2-Bottle Choice protocol (IA2BC, details can be found below) without the CRS; Group 2 stress exposure (*EtOH−/CRS+*, *n* = 20, 10 females) included animals that were not in the IA2BC but had the CRS protocol; Group 3 ethanol and stress exposure (*EtOH+/CRS+*, *n* = 28, 14 females) included animals under both the IA2BC and CRS protocols; and Group 4 control (*EtOH−/CRS−*, *n* = 20, 8 females) consisted of animals with no interventions (Fig. 1A). For Group 4, two bottles were presented with only water to control for the IA2BC conditions. Each rat was designated as the experimental unit and was considered as a random effect for the statistical analysis. The number of subjects per group is comparable with the largest sample sizes reported in the literature for deformation-based morphometry and resting-state functional magnetic resonance imaging (rsfMRI) in rodents.^39-41^

All experimental procedures, animal provenance and care were carried out in accordance with the ‘Reglamento de la Ley General de Salud en Materia de Investigación para la Salud’ (Health General Law on Health Research Regulation) of the Mexican Health Ministry, which follows the ‘Guide for the care and use of laboratory animals’^42^ and the ‘Norma Oficial Mexicana’ (NOM-062-ZOO-1999). The animal research protocols performed were approved by the ethical committee of the Neurobiology Institute of the National Autonomous University of Mexico, project number A113. Appropriate procedures were performed to minimize animal suffering. This study adhered to the ARRIVE 2.0 guidelines for reporting animal research.^43^

### Intermittent access 2-bottle choice

The intermittent access to 20% ethanol in a 2-bottle choice procedure (IA2BC) protocol was adapted from the original protocol established by Simms *et al*.,^44-47^ (Fig. 1B). The IA2BC has been shown to induce a gradual escalation of levels of voluntary ethanol consumption that reach pharmacologically relevant blood ethanol concentrations (BECs).^15,44^ The escalation has been shown to model the transition from low drinking to excessive alcohol consumption and increase the binge drinking.^15^ In this protocol, rats could consume water or ethanol (20%) on Monday-Wednesday-Friday. Twenty-four hours after placement, the EtOH bottle was weighted to obtain the mean of the intake along the 24-hours (g/kg/24-hour units), substance preference measures, and the binge (g/kg/30-min units) used as a measure of initial phase of AUD. To avoid non-intended liquid dropping, the anti-drop bottles were filled with parafilm. Prior to conducting the statistical group differences analysis, extreme values were identified and removed using the interquartile range method. Specifically, values above Q3 + 3x IQR or below Q1 − 3x IQR. Outlier values (above Q3 + 1.5xIQR or below Q1 − 1.5x IQR) were retained based on the previous findings indicating the presence of high and low ethanol intake patterns.^16^ Details about the description and procedures can be found in Supplementary Materials: Methods and in the workgroup’s repository.^48^

### Chronic restraint stress (CRS)

The chronic stress protocol was conducted using movement restraint as a stressor according to established protocols.^49^ Movement restriction was performed intermittently for three hours from 11:00 (±30 min) to 14:00 (±30 min), five times per week. The stress days were selected randomly in order to diminish the habituation process.^50^ The motion restrictor was constructed of acrylic with a double adjustment for the size of the rat (Fig. 1C). All rats were weighed daily as a measure of stress response as well as food consumption.^49^ Weight normalization was performed by subtracting the weight at P45 from all weight values according to age to obtain the weight change.

### Blood corticosterone concentration

A representative subsample of animals was randomly selected (*n* = 21, 9 females) to reflect the distribution across experimental groups and sex. Blood samples were obtained from the rat tail at the end of the protocol (P142) in order to validate and verify CRS-induced increase in corticosterone levels, as have been previously reported^51^ (Fig. 1). Corticosterone quantification was performed using a competitive enzyme-linked immunosorbent assay (ELISA) specifically designed to mouse and rats samples (Cat. No. 55-CORMS-E01, ALPCO) following the manufacturer's instructions. Additional details are provided in Supplementary Materials: Methods.

### Experimental design

The experimental design is outlined in Fig. 1D. The protocol was divided into four phases, with two main intervention phases: first exposure and reinstated phase. 1) Baseline, in which animals underwent initial behavioural testing (Behav-T1) at P36, followed by the first MRI acquisition (MRI-T1) at P44 ± 1 day. In the initial exposure phase, rats were randomly assigned to one of four groups for a 45-day period (20 sessions of alcohol and/or 30 days of CRS). Additional MRI acquisitions were performed during this phase at P67 ± 1 day (MRI-T2) and at P91 ± 1 day (MRI-T3). The third phase, designated as the withdrawal phase, commenced with the cessation of alcohol intake and the implementation of chronic restraint from P91 to P110. During this period, a second behavioural test was conducted, commencing at P100. In the reinstated phase, at P111, both interventions were implemented once more. A final MRI acquisition was made at the conclusion of the phase, at P145 ± 1 day (MRI-T4). Finally, the animals were euthanized (P146), and the brain was extracted for immunohistochemistry purposes, which will be analysed in a subsequent study.

### Behavioural tests

To explore if the effects of stress have long-lasting effects on anxiety-like behaviour and recognition memory, the animal's behaviour during the withdrawal phase was evaluated using the elevated plus maze (EPM) and novel object recognition (NOR) tasks. We also evaluated the preference for alcohol over water using the place preference test (CPP). All behavioural tests were conducted under environmental conditions similar to those of the housing but with dim red lighting. The videos were preprocessed using ffmpeg to ensure consistent dimensions and object positions. DeepLabCut v.2.3.8^52^ was employed for automated behavioural tracking for EPM and NOR (network training details can be found in the Supplementary Materials: Methods). Data tracking generated by DeepLabCut tracking was further processed using DLCAnalyzer^53^ in R programming language (see Code Availability section for details). Details of the statistical analysis can be found in the Supplementary Materials: Methods.

### Magnetic resonance imaging

Neuroimaging data was obtained at the National Laboratory for Magnetic Resonance Imaging (LANIREM) using a 7-Tesla Magnetic Resonance Imaging (MRI) scanner (Bruker Pharmascan 70/16 US) with a 2 × 2 array surface rat head coil. Structural MRI (sMRI) was acquired using a 3D FLASH T2-weighted sequence with TR = 30.76 ms, TE = 5 ms, flip angle = 10°, FOV = 25.6 × 19.1 × 25.6 mm, and isotropic resolution of 160 μm. Resting state functional MRI (rsfMRI) data were collected using a GE-EPI sequence with TR = 1000 ms, TE = 20 ms, flip angle = 60°, slice thickness = 1 mm, FOV = 30 × 30, number of slices = 24, and 600 volumes.

All MRI scans were converted from Bruker format to NIfTI using the brkraw toolbox v0.3.11^54^ within the BIDS framework.^55^ Structural T2w images underwent preprocessing including intensity normalization,^56^ image centering, and denoising via an in-house pipeline based on MINC-toolkit-v2 and ANTs tools.^57^ Deformation Based Morphometry (DBM) using a Two-Level approach with the SIGMA template v.1.2.1^58^ was applied for volume analysis. Voxel-wise longitudinal volume changes were analysed, treating relative Jacobian determinants as dependent variables and age, group, and sex as independent variables. MRI brain volume was linked to behavioural outcomes using partial least squares (PLS) correlation. For this analysis we associated the MRI-T3 with Behav-T2 acquisition points as they were acquired in adjacent time points. Functional rsfMRI data underwent preprocessing with the RABIES pipeline.^59^ Subnetworks were identified via NBR with linear mixed models,^60,61^ and functional connectivity trajectories were analysed for significant group differences. Whole details can be found in Supplementary Materials: Methods.

### Statistical analysis

All statistical tests were performed through linear mixed-models or linear models in the case of novel object recognition. The local volume, functional correlation, weight change, ethanol intake, anxiety index, and preference index were considered as dependent variables. The group session and the sex of the subjects were included as interaction fixed effects (independent variables), and rat identification (RID) was included as a random effect. Batch was utilized as a covariate in all models. Conversely, the discrimination ratio was analysed by employing a linear regression model (see Supplementary Materials: Methods, Eq. 6). Standardized parameters were obtained by fitting the model on a standardized version of the dataset. The 95% confidence intervals (CIs) and *P*-values were computed using a Wald t-distribution approximation. Estimated marginal means (EMMs) were calculated for the purpose of making comparisons between groups, including pairwise post-hoc tests and contrasts.


(1) Metric∼Group⋅Age⋅Sex+Batch+(1|RID)

Metrics: local volume, functional connectivity, ethanol main intake, *Δ* weight = (weight in each session—weight of the first session), anxiety index, and preference index.

For all analyses, the alpha level was set at 5%. False-discovery rate (FDR) analysis at q = 0.05 was conducted to adjust for multiple comparisons. All analyses were performed on R-programming language v.4.1. Scripts necessary to reproduce the analysis and ROI-specific masks are available in the GitHub repository (see code availability section).

## Results

### Ethanol intake

**Figure 2:**
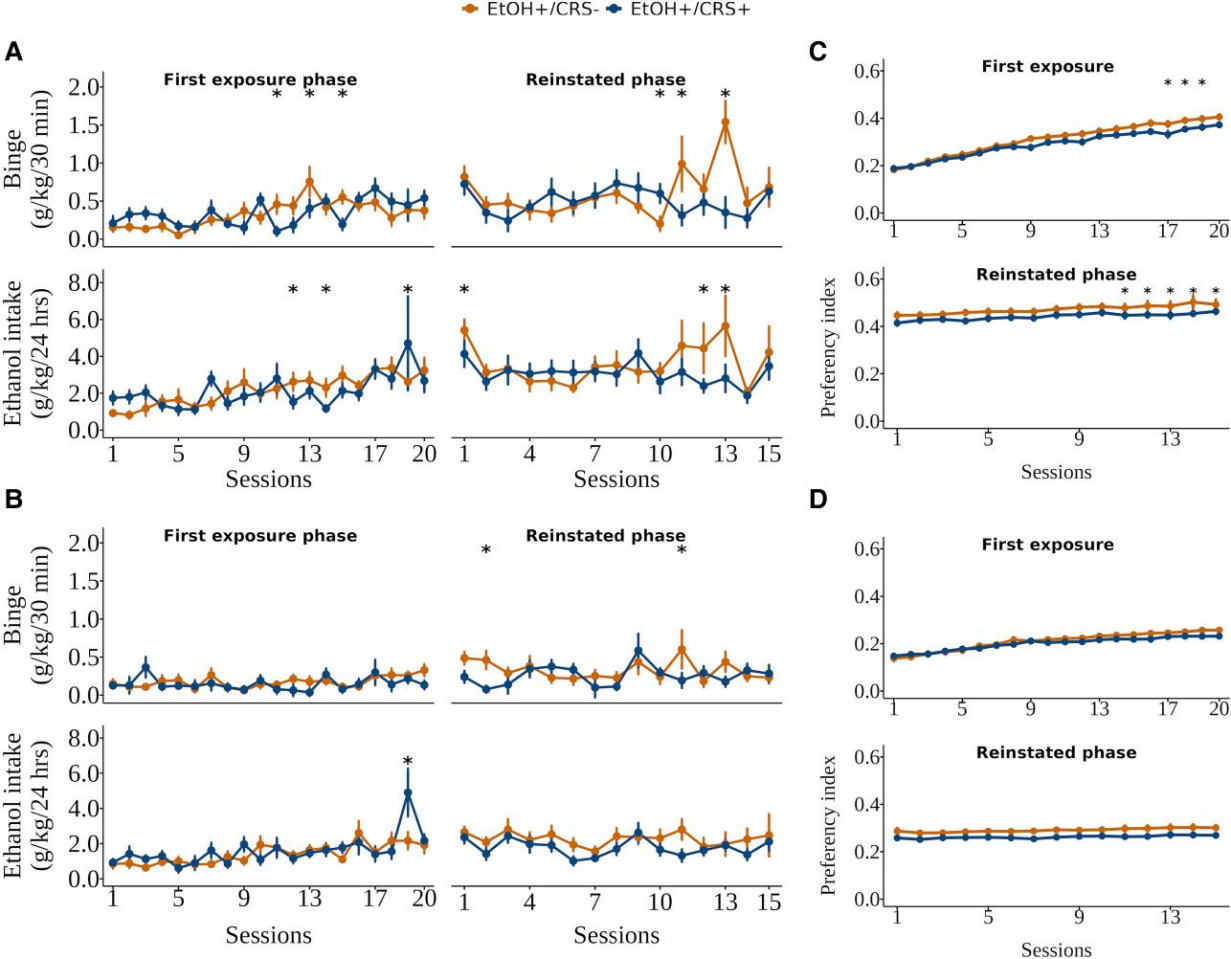
**Mean of the ethanol intake and preference.** Chronic stress reduces ethanol intake and CPP in only a few sessions in males. Mean intake and standard error for (*n* = 28 animals per session per session for both EtOH+ groups, extreme values were excluded) (**A**) Male and (**B**) female binge and ethanol intake over the twenty sessions of the first exposure and in the fifteen sessions of the reinstated phase (See Fig. 1). Ethanol preference index of ethanol over water for (*n* = 28 animals per session per session for both EtOH+ groups, extreme values were excluded) (**C**) Male and (**D**) female, the threshold above 0.5 means EtOH preference index. Experimental unit: individual ethanol intake with rat-ID as a random effect. Group differences were assessed by a linear mixed model, formula: ethanol/binge intake ∼ Group·Age·Sex + Batch + (1|RID). Extreme values (above Q3 + 3x IQR or below Q1 − 3x IQR) were removed based on the interquartile range (IQR). Estimates, confidence intervals and associated q-values can be found in Supplementary Table 1. Abbreviations: EtOH, ethanol intake; CRS, chronic restraint stress.

The significant pairwise comparisons of the IA2BC model indicated specific group differences in the main intake of ethanol across only some sessions (Fig. 2A and B). Chronic restraint stress (EtOH+/CRS+) reduced ethanol intake in males compared to the EtOH+/CRS− group in only five sessions, in contrast with one session in females. A transient increase was observed in the CRS group on the final day of the reinstated phase; however, averaging the last three sessions showed no group differences compared to controls (F(3,32) = 3.138, β = −0.983 *P* = 0.429; females: *P* = 0.53; males: *P* = 0.13), reflecting daily fluctuations in alcohol consumption. Preference for ethanol over water was also diminished. The CPP task preference index also showed lower ethanol preference in the EtOH+/CRS+ male group (Fig. 2C and D). Interestingly, after the withdrawal period, the male EtOH+/CRS- group consumed larger quantities of ethanol than before (the rebound effect), which was not observed in the EtOH+/CRS+ male group or the two female groups. In both groups, we observed higher ethanol intake in males than in females, primarily during the reinstated phase (Supplementary Table 4). Estimates, confidence intervals and associated q-values can be found in Supplementary Table 1.

### Weight

**Figure 3:**
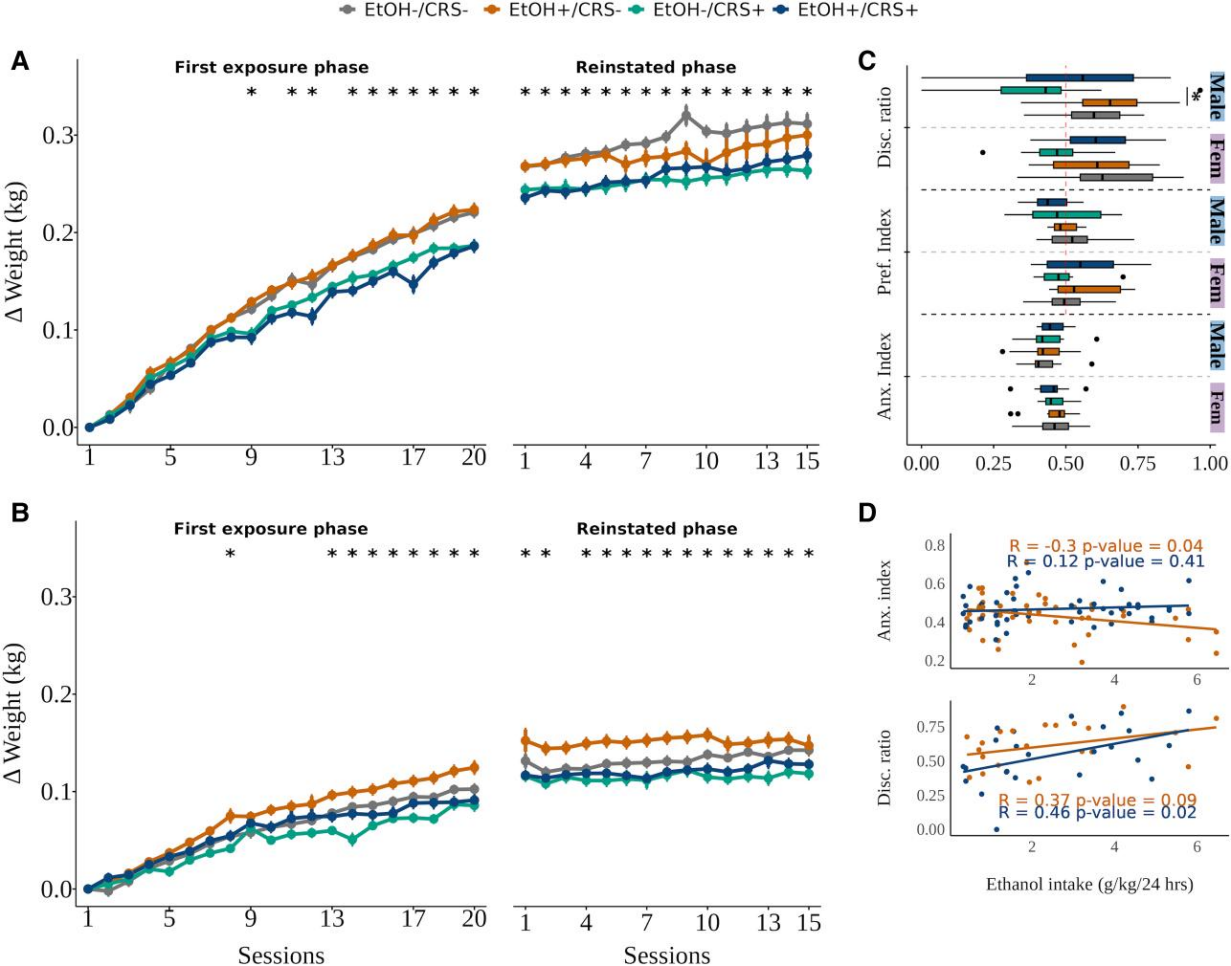
Body weight gain and behaviour. Chronic stress reduces body weight gain and affects object recognition memory in males. (**A**) male and (**B**) female weight change (kg) over the twenty sessions of the first exposure and in the fifteen sessions of the reinstated phase (*n* = 28 animals per session per session for both EtOH+ groups and *n* = 20 for EtOH−/CRS+ and EtOH−/CRS− groups, extreme values were excluded). Estimates, confidence intervals and associated q-values can be found in Supplementary Table 2. (**C**) Indexes from the behavioural evaluation (Behav-T2, *n* = 28 animals per session per session for both EtOH+ groups and *n* = 20 for EtOH−/CRS+ and *n* = 19 for EtOH−/CRS− group). Significant value in discrimination ratio (β = 0.26, q = 0.023, *P* = 0.003, d = 1.53). (**D**) Pearson correlation between EPM (Anxiety Index, *n* = 28 animals per session per session for both EtOH+ groups) and NOR (Discrimination ratio, *n* = 27 animals per session per session for EtOH+/CRS− and *n* = 26 animals per session for EtOH+/CRS+ group) metrics with the average of ethanol intake across sessions of the first phase. Abbreviations: Anxiety index = calculated based on elevated plus maze task (Supplementary Materials: Eq. 2). Disc. ratio = Discrimination ratio calculated based on novel object recognition task (Supplementary Materials: Eq. 3), threshold above 0.5 means novel object interaction. Pref. Index = Preference index calculated based on conditioned place preference task (Supplementary Materials: Eq. 4), threshold above 0.5 means ethanol chamber preference. Experimental unit: individual weight/behaviour indexes with rat-ID as a random effect. Group differences were assessed by a linear mixed model, formula: weight ∼ Group·Age·Sex + Batch + (1|RID). Extreme values (above Q3 + 3x IQR or below Q1 − 3x IQR) were removed based on the interquartile range (IQR).

During the sessions, the body weight of animals under CRS (EtOH−/CRS+, EtOH+/CRS+) decreased compared to the control and ethanol intake groups, even during the relapse phase (Fig. 3A and B). Estimates, confidence intervals, and associated q-values can be found in Supplementary Table 2. See the Supplementary Materials: Results for food consumption throughout the protocol.

### Blood corticosterone concentration

As expected, corticosterone levels in blood serum were significantly higher in both CRS-exposed groups compared to the non-stressed control group (EtOH−/CRS+: q = 0.006; EtOH+/CRS+: q = 0.004) and with the EtOH+/CRS− group (EtOH-/CRS+: q = 0.013; EtOH+/CRS+: q = 0.006). These results support the effectiveness of the CRS protocol in increasing corticosterone levels (See Supplementary Materials: Results).

### Behavioural test

The anxiety index obtained from the EPM task did not show any differential performance depending on the treatment (q > 0.05) or sex (q > 0.05), meaning none of the groups exhibited overall anxiety-like behaviours after chronic stress or chronic ethanol intake (Fig. 3D). However, individual EPM metrics (Supplementary Materials: Results) revealed that female rats (EtOH+/CRS− and EtOH+/CRS+) exposed to chronic ethanol intake exhibited anxiety-related behaviours, such as spending more stationary time during the task and moving more slowly than the female control group. Nevertheless, the anxiety index (a global measure) did not differ between groups or sexes.

The object recognition of the NOR task was lower of novel objects only in male EtOH−/CRS+ rats compared to male EtOH+/CRS− rats (β = 0.26, q = 0.023, *P* = 0.003, d = 1.53), and uncorrected compared with male rats of the EtOH−/CRS− (β = 0.19, q = 0.096, *P* = 0.048, d = 1.1) and EtOH+/CRS+ groups (β = −0.17, q = 0.096, *P* = 0.048, d = −0.97) (Fig. 3D). In contrast, individual measures (See Supplementary Materials: Results) showed that the EtOH+/CRS+ group had lower exploration metrics of the novel object (covering more distance, reaching higher speeds, and moving for longer periods of time) than the EtOH+/CRS− group. In summary, males exposed to chronic stress showed lower novel object recognition than males exposed to chronic ethanol intake and male controls.

### Voxelwise analysis

**Figure 4:**
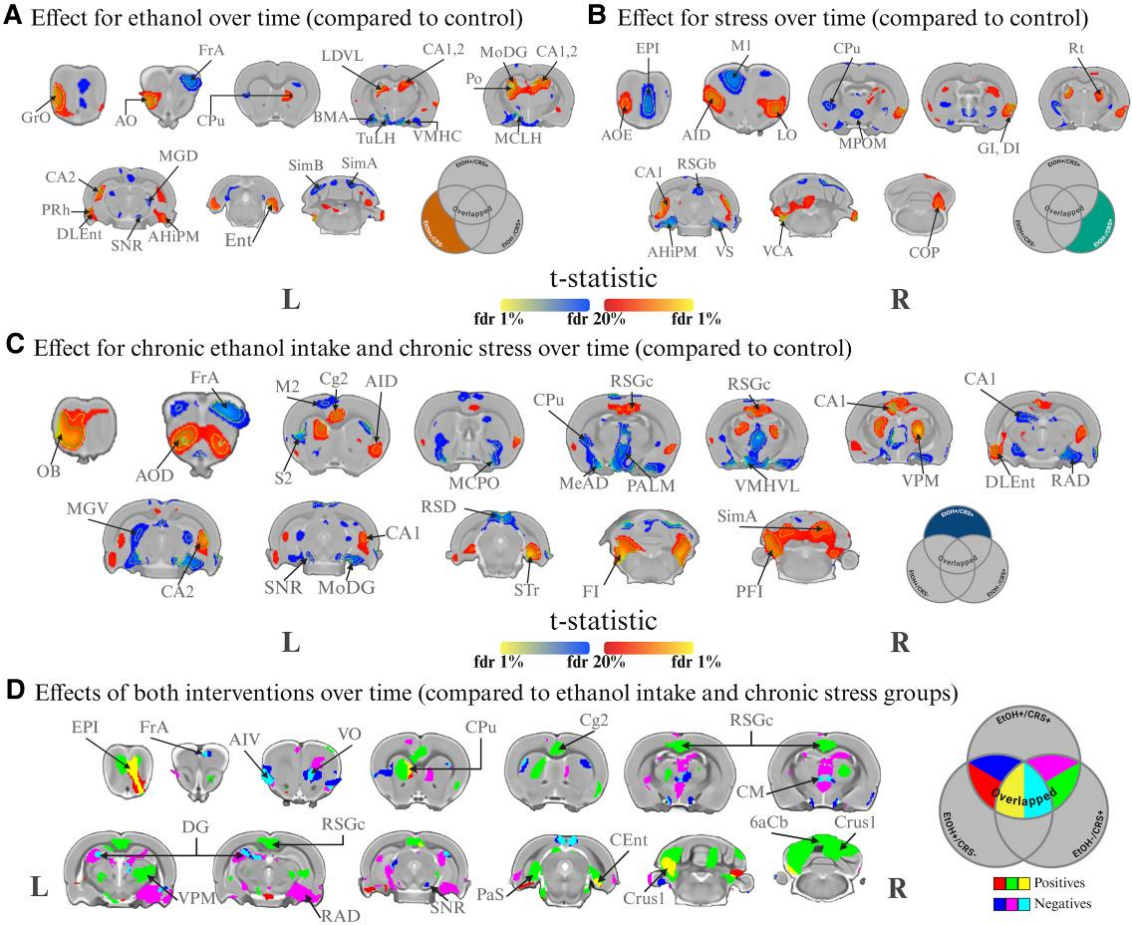
Structural effects of chronic ethanol intake, chronic restraint stress and both interventions among the control group (interaction age*group*sex). Chronic ethanol intake and chronic stress affected more extended areas contrasted with all groups. The effect appear to be additive (yellow and light blue) in olfactory bulb (EPI), ventral orbitofrontal cortex (VO), dorsal caudate-putamen (CPu) and dentate gyrus (DG) and cerebellum (Crus 1). (**A**) Longitudinal structural effects of chronic ethanol intake (EtOH+/CRS−), (**B**) chronic stress (EtOH−/CRS+) and (**C**) the effects of both interventions (EtOH+/CRS+) over the control (EtOH−/CRS−). To explore the additive effect of each intervention in the EtOH+/CRS+ group, we contrasted this group against EtOH+/CRS− or EtOH−/CRS+ (**D**) Additive effect of chronic ethanol intake (EtOH+/CRS + > EtOH−/CRS+; red: positive t-values, blue: negative t-values), chronic stress (EtOH+/CRS + > EtOH+/CRS−; green: positive t-values, purple: negative t-values) and overlapping effects of both. Colour map describes the direction of the t-statistics; cooler colours denoting negative values; most commonly corresponding to volume decline and warmer colours denoting positive values. Experimental unit: individual jacobian determinant with rat-ID as a random effect (*n* = 28 animals per session per MRI session for both EtOH+ groups and *n* = 20 for EtOH−/CRS+ and EtOH−/CRS− groups). Group differences were assessed by a linear mixed model, formula: jacobian determinants ∼ Group·Age·Sex + Batch + (1|RID). Threshold set at FDR of 20%, 5% (yellow dashed line) and 1% (green dashed line). Coronal slides labels according to Paxinos & Watson stereotaxic atlas.^62^ Abbreviations: 2Cb, 2nd cerebellar lobule; 6aCb, 6a cerebellar lobule; 8Cb, 8th cerebellar lobule; 9Cb, 9th cerebellar lobule; AHiPM, amygdalohippocampal area, posteromedial part; AID, agranular insular cortex, dorsal part; AIV, agranular insular cortex, ventral part; AO, anterior olfactory area, dorsal part; AOD, anterior olfactory area; AOE, anterior olfactory nu, external; Apir, amygdalopiriform transition area; BMA, basomedial amygdaloid nucleus, anterior part; CA1, hippocampus, CA1; CA2, hippocampus, CA2; Cent, caudomedial entorhinal cortex; Cg2, cingulate cortex, area 2; CM, central medial thalamic nucleus; COP, copula of the pyramid; CPu, caudate-putamen (striatum); Crus1, crus 1 of the cerebellum; DG, dentate gyrus; DI, dysgranular insular cortex; DLEnt, dorsal intermediate entorhinal cortex; Ent, entorhinal cortex; EPI, external plexiform layer of the olfactory bulb; FI, flocculus; FrA, frontal association cortex; GI, granular insular cortex; GrA, granule cell layer of the accessory olfactory bulb; GrO, granular cell layer olfactory bulb; LDVL, laterodorsal thalamic nucleus, ventrolateral part; LO, lateral orbital cortex; M1, primary motor cortex; M2, secondary motor cortex; MCLH, magnocellular nucleus of the lateral hypothalamus; MCPO, magnocellular preoptic nucleus; MeAD, medial amygdaloid nucleus, anterodorsal part; MGD, medial geniculate nucleus, dorsal part; MGV, medial geniculate nucleus, ventral part; MoDG, molecular layer of the dentate gyrus; MPOM, medial preoptic nu, medial; OB, olfactory bulb; PALM, paraventricular hypothalamic nucleus, lateral magnocellular part; PaS, paratrigeminal nucleus; PFI, paraflocculus; Po, posterior thalamic nuclear group; PRh, perirhinal cortex; PrL, prelimbic cortex; RAD, radiatum layer of the hippocampus; Re, reuniens thalamic nucleus; RSD, retrosplenial dysgranular cortex; RSGb, retrosplenial granular cortex, b region; RSGc, retrosplenial granular cortex, c region; Rt, reticular thalamic nucleus; S2, secondary somatosensory cortex; SimA, simple lobule A; SimB, simple lobule B; SNR, substantia nigra, reticular part; STr, superior thalamic radiation; TuLH, tuberal region of lateral hypothalamus; V1, primary visual cortex; V2, secondary visual cortex; VCA, ventral cochlear nucleus, anterior part; VMHC, ventromedial hypothalamic nucleus, central part; VMHVL, ventromedial hypothalamic nucleus, ventrolateral part; VO, ventral orbital cortex; VPM, ventral posteromedial thalamic nucleus; VS, ventral subiculum.

Chronic ethanol intake and chronic stress (EtOH+/CRS+) had an additive effect on cortical and subcortical brain regions, including the olfactory bulb (OB), frontal association area (FrA), secondary motor area (M2), secondary cingulate cortex (Cg2), entorhinal cortex (Ent), cerebellum (Cer), caudate-putamen (CPu), hippocampus, and thalamic areas (Fig. 4A). Compared with the ethanol intake (EtOH+/CRS−) group, this group (EtOH+/CRS+) displayed volume reductions in the frontal associative cortex (FrA), insular cortex (AIV), ventral orbitofrontal cortex (VO), and retrosplenial cortex (RSGc). Increased volumes were found in the olfactory bulb (OB), entorhinal cortex (Ent), caudate-putamen (CPu), and cerebellum (Fig. 4B). Conversely, when compared to the EtOH−/CRS+ group, a decrease was observed in the FrA, VO, CPu, hippocampus, hypothalamus, and amygdala (Amy), while increased volumes were observed in the OB, CPu, Cg2, RSGc, Ent, cerebellum, and thalamic areas (Fig. 4B).

Overall, we found that male rats exposed to chronic stress and chronic ethanol intake exhibited greater longitudinal volume changes than all other groups, including females. These effects appear to be additive in the olfactory bulb (OB), ventral olfactory cortex (VO), caudate putamen (CPu), dentate gyrus (DG) of the hippocampus, and crus 1 of the cerebellum when compared with groups that experienced only one intervention and females. In general, males are more affected than females in terms of local volume.

### Structural brain volume and behaviour relationship

**Figure 5:**
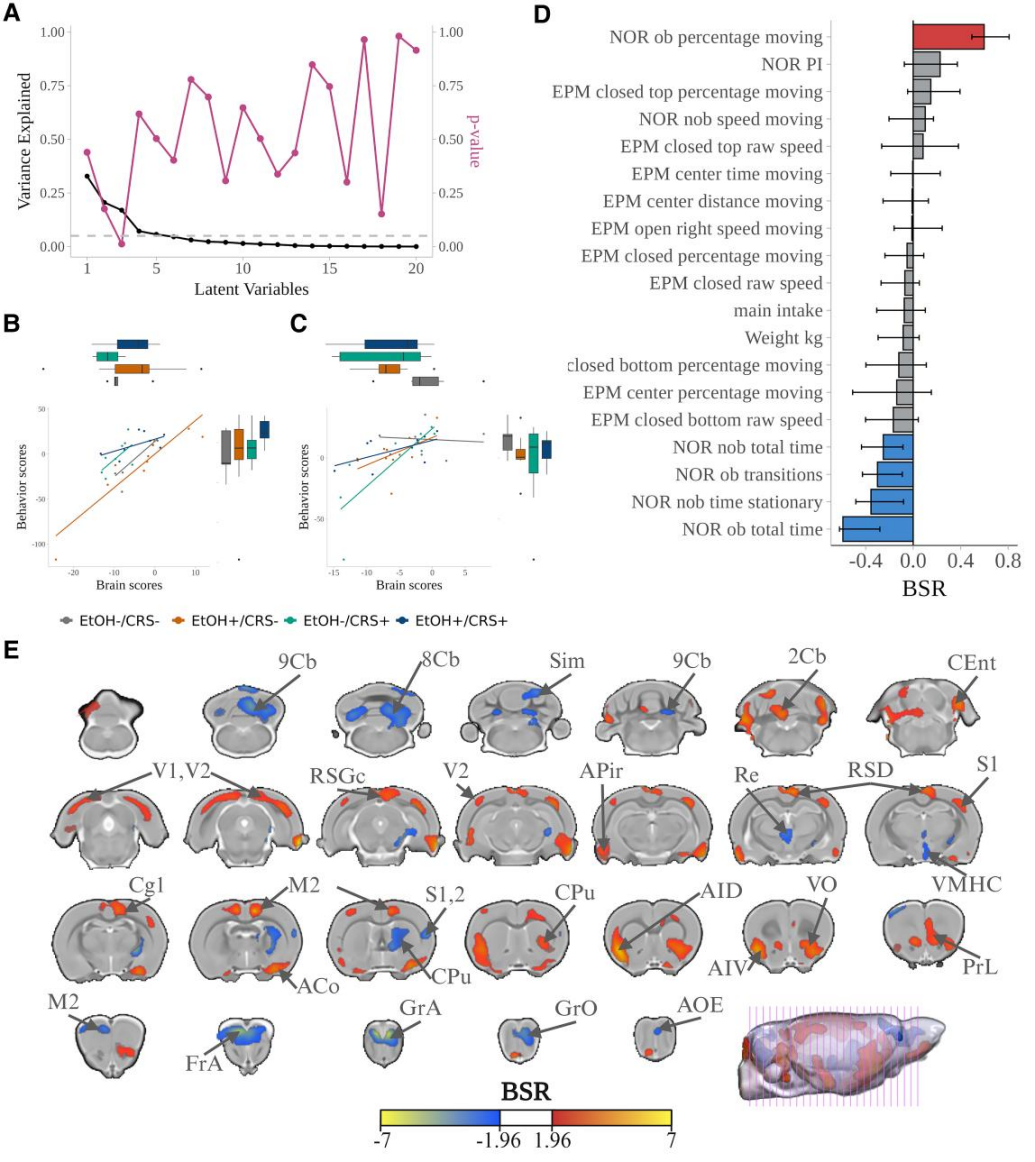
**Relationship between the brain volume and EPM-NOR-behaviour of the latent variable 3 (LV3).** (**A**) The third LV explained 17% of the covariance between brain and behaviour metrics (*P* = 0.011, evaluated using permutation test; contribution of variables assessed with bootstrap resampling). Dashed line at 0.05. Pairwise group comparisons of the PLS brain and behaviour scores of LV3 on (**B**) males and (**C**) females. Experimental unit: individual brain/behaviour score with rat-ID as a random effect (Anx. Index, *n* = 28 animals per session per session for both EtOH+ groups; Disc. ratio, *n* = 27 animals per session per session for EtOH+/CRS− and *n* = 26 animals per session for EtOH+/CRS+ group). Group differences were assessed by a linear mixed model, formula: alcohol intake ∼ Group·Age·Sex + Batch + (1|RID). *q < 0.05, **q < 0.01, ***q < 0.001. (**D**) Spatial brain patterns of the contribution to the bootstrap ratio (BSR) values. Cold colours indicate negative BSR values, while warm indicates positives (threshold set to 1.96). (**E**) Behavioural metrics patterns associated with the brain pattern. Bars are coloured to show significant association. The brain pattern is associated with moving-related metrics in positive BSR values, while negatives are associated with memory-related metrics. Coronal slides labels according to Paxinos & Watson stereotaxic atlas.^62^ Abbreviations: NOR-ob, familiar object, NOR-nob, novel object, PI, preference index, 8Cb, 8th cerebellar lobule; 9Cb, 9th cerebellar lobule; Sim, simple lobule; 2Cb, 2nd cerebellar lobule; Cent, caudomedial entorhinal cortex; V1, primary visual cortex; V2, secondary visual cortex; RSGc, retrosplenial granular cortex, c region; Apir, amygdalopiriform transition area; Re, reuniens thalamic nucleus; RSD, retrosplenial dysgranular cortex; S1, primary somatosensory cortex; Cg1, cingulate cortex, area 1; M2, secondary motor cortex; S2, secondary somatosensory cortex; CPu, caudate-putamen (striatum); AID, agranular insular cortex, dorsal part; AIV, agranular insular cortex, ventral part; VO, ventral orbital cortex; PrL, prelimbic cortex; FrA, frontal association cortex; GrA, granule cell layer of the accessory olfactory bulb; GrO, granular cell layer olfactory bulb; AOE, anterior olfactory nu, external.

The partial least squares (PLS) analysis revealed one significant latent variable (LV-3), which explained 17% of the variance (*P* = 0.011). The other components accounted for most of the variance, but were not significant (Fig. 5A). Post hoc analysis revealed no group differences in behaviour or brain metrics (Fig. 5B and C). Brain-behaviour correlations included associations between locomotor activity (NOR moving time percentage) and volumes of the prelimbic, orbitofrontal, insular, motor, primary cingulate, somatosensory, visual cortices, ventral striatum, and cerebellum. Transitions and total and stationary time in the NOR were associated with the frontal, associative, and somatosensory cortices; the striatum; the hypothalamus; the thalamus; and the cerebellar subregions (Fig. 5D and E). PLS findings indicated significant associations between locomotor activity and cortical regions, as well as between recognition memory metrics with subcortical and cerebellar regions, in all groups.

### Functional connectivity

**Figure 6:**
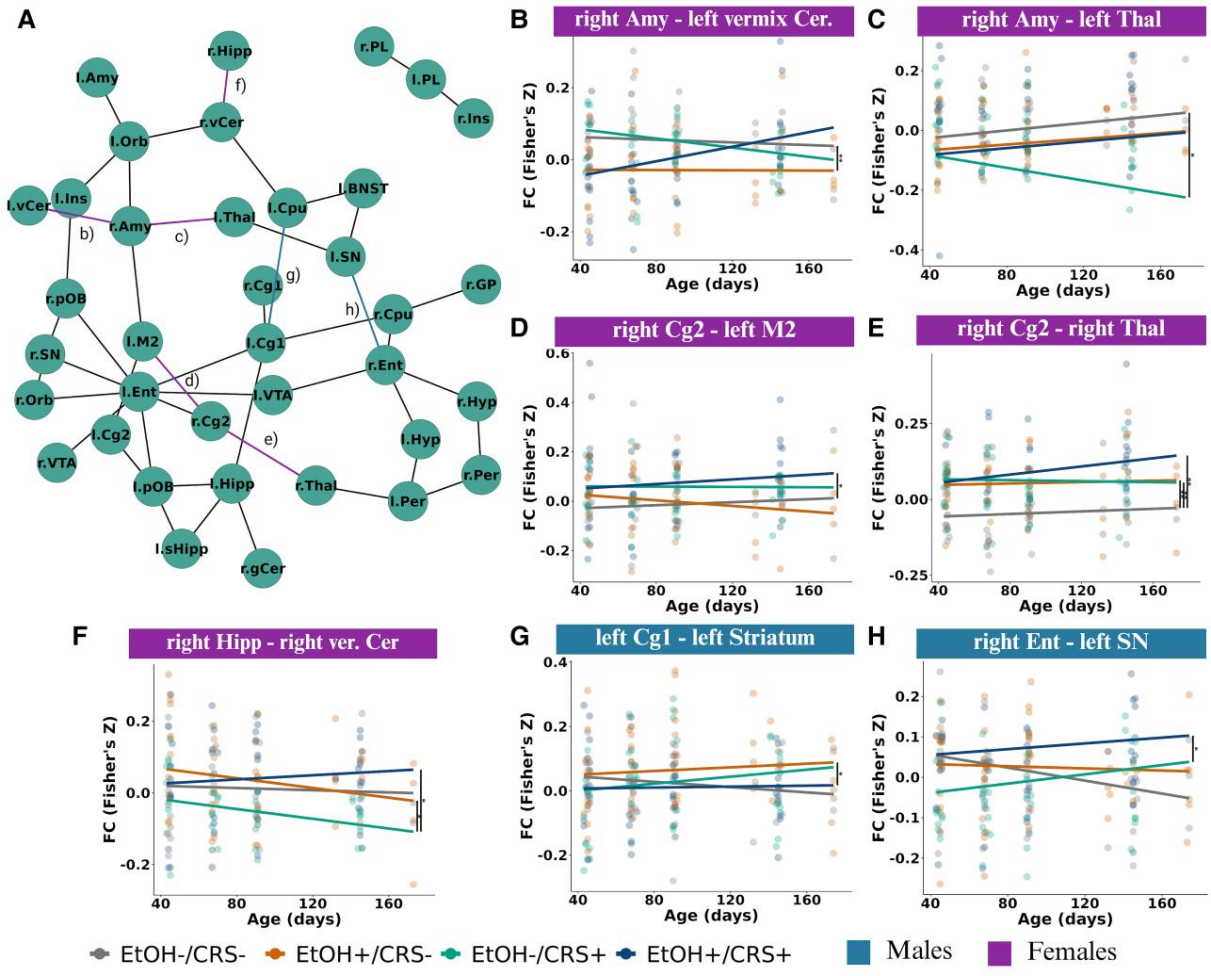
Functional connectivity trajectories of chronic ethanol intake and chronic restraint stress along the whole protocol (interaction age*group*sex). Chronic stress altered the functional connectivity of 37 regions compared with the control group, while both interventions (EtOH+/CRS+) changed the FC of more connections over the neurodevelopment. (**A**) NBR analysis via linear mixed model analysis results in regions (nodes) comprising the brain network with significant changes in functional connectivity (Fisher z-transformed correlation values) along the age. **B–H**) Scatter plot (residuals) with regression lines showing the trajectory of each connection. Abbreviations: secondary cingulate cortex (Cg2), secondary motor cortex (M2), primary cingulate cortex (Cg1), entorhinal cortex (Ent), hippocampus (Hipp), thalamus (Thal), amygdala area (Amy), substantia nigra (SN). Experimental unit: individual FC with rat-ID as a random effect (*n* = 28 animals per session per MRI session for both EtOH+ groups and *n* = 20 for EtOH−/CRS+ and EtOH−/CRS− groups). Group differences were assessed by a linear mixed model, formula: functional connectivity ∼ Group·Age·Sex + Batch + (1|RID). Estimates, confidence intervals and associated q-values can be found in Supplementary Materials: Results. Abbreviations: r, right; l, left; gaOB, Glomerular Layer of the Accessory Olfactory Bulb; gCer, Granule Cell Level of the Cerebellum; glOB, Glomerular Layer of the Olfactory Bulb; GP, Globus Pallidus; Hipp, Hippocampus; Hyp, Hypothalamic Region; Ins, Insular Cortex; lCer, Lateral Molecular Layer of the Cerebellum; M1, Primary Motor Cortex; M2, Secondary Motor Cortex; Orb, Orbitofrontal cortex; Per, Perirhinal Area; PL, Prelimbic System; pOB, Posterior Olfactory Bulb; Ret, Retrosplenial Cortex; sHipp, Subiculum; SN, Substantia Nigra; Thal, Thalamus; vCer, Vermix Molecular Layer of the Cerebellum; VTA, Ventral Tegmental Area.

The NBS linear mixed-effects analysis (Supplementary Materials: Equation 7) revealed significant effects in the EtOH−/CRS+ group compared to the EtOH−/CRS− group. This analysis identified 46 connections across 37 regions (q = 0.032; Fig. 6A). Subsequent post hoc testing of significant group differences in functional connectivity identified seven altered edges involving the amygdala, thalamus, caudate-putamen, substantia nigra, hippocampus, cerebellum, entorhinal cortex, secondary motor cortex, and primary and secondary cingulate cortex (Figs 6B–H).

Chronic ethanol intake alone altered functional connectivity (FC) in only one region (the right amygdala and the cerebellum), while chronic stress alone or in combination with ethanol intake altered FC in more regions compared to the control group. Female rats exhibited altered connectivity patterns between the right amygdala and the vermis of the cerebellum, the thalamus, the hippocampus, and the secondary cingulate cortex and motor cortex (Fig. 6B–F). Males showed increased connectivity in cortico-subcortical regions (Fig. 6G and H), and the effects of stress were more pronounced in females than in males. Overall, we found that chronic stress altered functional connectivity in 37 cortical and subcortical regions compared to the control group. The combination of both interventions increased connectivity, mostly in cortical-to-subcortical connections (Fig. 6D–H and Supplementary Table 3). This effect was more pronounced in females than in males, which was the opposite of the structural results.

## Discussion

In the present study, we examined the effects of chronic stress on ethanol intake, as well as its implications for behavioural tasks, brain volume, and functional connectivity. Our findings revealed that chronic stress exerted no significant impact on ethanol intake during the initial 20 sessions. However, we observed alterations in corticosterone levels and recognition memory index. Neuroimaging analysis revealed significant neuroanatomical changes in males across several brain regions due to chronic stress and ethanol intake, with additive effects observed in the olfactory bulb, caudate-putamen, orbitofrontal, hippocampus, and cerebellum. Notably, these alterations manifested to a greater extent in female subjects, particularly within networks such as the amygdala-thalamus and cingulate-striatum.

Contrary to the findings of some animal studies, our research did not demonstrate a heightened ethanol intake in response to chronic stress.^10^ However, we observed a tendency for a decrease in ethanol intake after 31 sessions. A body of previous research has addressed the incongruent outcomes observed in acute and subchronic stress studies. A number of studies have been conducted on the relationship between stress and ethanol intake. The results of these studies have been conflicting, with some finding that stress increases ethanol intake, while others have found that stress reduces ethanol intake. Nonetheless, the majority of studies have reported a lack of significant outcomes.^10^

In the present study, it was observed that chronic stress diminished ethanol intake in two sessions of the reinstated phase, exclusively in male subjects, suggesting a possible dynamic relationship between both conditions that cannot be studied in short time frames. Session-to-session variability is common in the IA2BC model,^15^ and the transient increase observed on the final day of the reinstated phase did not persist when averaging across sessions, confirming it as a fluctuation rather than a consistent effect. However, it is possible that with longer exposure such fluctuations could evolve into a stable trend, which should be explored in future studies. Chronic stress may play a role in ethanol intake in the latter stages. The age of onset of chronic stress and/or ethanol intake may also be a contributing factor. The onset of either condition in adolescence has been shown to differ from the onset in adulthood. Our study is also the first one to investigate chronic stress and chronic ethanol intake together from adolescence to adulthood. Therefore, our results are novel and should be further replicated, specifically testing the onset age of either or both conditions, even more in light of recent findings regarding the individual differences in coping with distress.^12,63^ The identification of vulnerable individuals through operant schedules tests^12^ or identifying subgrouping alcohol-drinking patterns^16^ could help to understand the stress-induced drinking effect. Finally, our research also indicated that males exhibited a higher level of ethanol consumption compared to females, predominantly during the reinstated phase.^64,65^ This result supports the idea that sex is related to the vulnerability to AUD but is also age onset and abstinence -dependent.^37^

Voxel-wise local volume analysis revealed region-specific changes in response to chronic ethanol intake, chronic stress, or their combination, highlighting distinct neurodevelopmental trajectories in rats that appear to be sex and age onset-dependent. These changes overlapped within the caudate putamen, the dorsal hippocampus, and the cerebellum—all of which are critical regions of associated with the effects of ethanol or stress.^9^ Some studies have suggested additive effects of chronic stress and ethanol due to cellular degeneration,^66,67^ but this has not been directly tested in animal models. The results of the functional connectivity (FC) analysis demonstrated a disruption in the integration of cortical and subcortical regions, including the cingulate-striatum, thalamus, and motor cortex. This disruption was particularly evident in female rats and was induced by chronic stress, thereby corroborating previous reports that alterations in network integration across brain regions can be induced by stress.^30,31^ The additive effect of both interventions over core regions implicated in addiction and stress-related circuitry could serve as potential target regions to understand the intricate mechanisms of stress effects over ethanol intake. The behavioural results indicated that chronic stress exerted an influence on locomotor activity and recognition memory, even in the absence of stressors (further discussion can be found in the Supplementary Materials: Discussion). Although volume-behavioural associations were non group specific, we found that the cortex/cerebellum volumes were linked to locomotor activity,^68-70^ as well as, the olfactory bulb and thalamus regions linked to recognition memory.^70,71^ Overall, the findings suggest that chronic stress may induce more widespread structural alterations in brain circuits associated with addiction and stress, potentially guiding future target regions for understanding stress effects on ethanol intake.

The present study assessed the longitudinal effects of chronic stress on ethanol intake, behaviour, and brain volume and connectivity from adolescence to adulthood. In this regard, the observed effects from all interventions were compared with the expected neurodevelopmental trajectories, in which measured metrics could be altered depending on the age of the intervention, as observed in alcohol consumption.^72^ Prior studies have demonstrated that rats of varying ages exhibit different responses to repeated stressors or alcohol-related behaviours. These variations can be attributed to disparities in the maturation of neuroendocrine and neurotransmission systems.^73-75^ In this context, both interventions have been observed to manifest distinct alterations in the neurodevelopmental trajectory, in addition to joint effects, as demonstrated by the comparison with the control group. Recent findings indicate that human adults exhibit altered brain volume due to alcohol intake across age.^21^ This observation suggests a potential interaction between alcohol consumption and the aging process, specifically regarding changes in brain structure.^21,76,77^ These results also could imply that both CRS and alcohol consumption may interfere with normal neurodevelopment at a macroscopic level, with the direction of these effects potentially varying depending on factors such as brain region, sex, and age at onset. This may underscore the intricate interplay of neuroplasticity processes during the maturation process. Further research is necessary to explore how these interventions interact with the natural aging process.

Sex differences are crucial in psychiatric studies because prior research has predominantly focused on males, excluding females and limiting our understanding of sex-specific mechanisms.^78^ In psychiatric research, sex effects have been suggested to be related to hormones. Due to gonadal hormone fluctuations, females have a different pattern of functionality in the HPA axis and LC-NA system, leading to different stress responsiveness.^5,38^ Females have more well-defined pulses or oscillations of stress-interacting hormones than males, which may be particularly relevant to the interaction between stress and ethanol intake.^79^ Human studies have demonstrated that, despite similar AUD prevalence, women consume less alcohol but may experience more severe effects.^36^ Early life stress is associated with problematic alcohol use in women and is linked to corticolimbic function, neuronal activity, inflammation, sex hormones, brain structure, and interhemispheric communication, all of which could alter brain function differently.^36,80^ Compared with males, our results showed fewer volume changes in females, which contrasts with altered functional connectivity. These findings suggest that chronic ethanol intake and stress may affect males and females differently in terms of behaviour, brain structure, and functional connections.^27,38,81^ Further research is needed to study this assumption using microstructural and other functional techniques to explore neuronal function and hormonal signalling pathways.^80^

## Limitations

The limitations include technical problems with the MRI scanner that resulted in the loss of seven images at the T2 and T3 time points. However, this did not affect the overall results because mixed model effects were used to account for the missing data. Additionally, the limited number of acquisition points and the absence of a post-withdrawal time point may have led to overinterpretation of the findings. Measuring blood corticosterone levels was valuable for validating the stress model; however, the analysis was performed on a representative subsample. Sampling all animals at multiple time points could have provided a more comprehensive understanding of HPA axis activity; however, the selected approach was sufficient to detect the expected group differences. For future studies, more refined techniques, such as liquid chromatography-mass spectrometry, could be useful. Furthermore, while the neuroimaging data were collected longitudinally, the behavioural data were collected cross-sectionally during an abstinence period. Future research should aim to extend these analyses across the lifespan. Finally, these findings should be interpreted with caution given the limitations of animal models for studying disorders. In this study, we used movement restraint only to model the complex phenomenon of stress-induced ethanol intake. This method may not fully capture the intricacies of this behaviour; however, it is considered a well-established animal model that mimics repeated stressful situations in humans, producing expected stress responses.^82,83^ In a similar vein, IA2BC has been identified as a pre-clinical model of alcohol abuse and dependence. This model has demonstrated the presence of individual differences, with approximately 20–30% of the total population exhibiting high levels of alcohol intake.^15,16^ This variability is reflected in the findings, which demonstrate higher variation in both 24-hour and binge metrics. The latter exhibited a higher consumption during the first 30-minutes in the EtOH+/CRS+ group that was not exhibited in the 24-hour intake. In the future, the use of larger sample sizes in studies of this nature, as well as the behavioural assessment of cue- and reward-driven behaviour could facilitate a more comprehensive comparison of the stress-induced effects associated with high and low alcohol intake patterns. In order to translate the present study to real-world ecological human conditions, it is necessary to expand the research to include different age-onset groups and broad stress protocols or a combination of them, such as the chronic variable stress protocol (CVS) or chronic unpredictable mild stress (CUMS).

## Conclusion

Overall, the present results reflect neuroadaptive or maladaptive neuroplastic responses especially with prolonged restraint stress, disrupting cognitive behaviour and altering the brain at macrostructural level, without any corresponding changes in ethanol intake levels. These results underscore the sex-dependent and region-specific effects of combined ethanol exposure and chronic stress on both the structural and functional networks of the brain, emphasizing the need for further studies to understand the mechanisms driving these interactions in the course of psychopathological development.

## Supplementary Material

fcaf432_Supplementary_Data

## Data Availability

The data that support the findings of this study are available on request from the corresponding author, E.A.G.V. For the code analysis, derivative metrics and extended results presented here, please check: https://github.com/JalilRT/Sudmex_alcohol_stress.
