# Supplementary material for: The effect of chronic stress and chronic alcohol intake on behaviour, brain volume, and functional connectivity in a longitudinal rat model
Source: Brain Commun. 2025 Nov 1;7(6):fcaf432. doi: 10.1093/braincomms/fcaf432 (PMC12624392; doi:10.1093/braincomms/fcaf432)
Supplement: fcaf432_Supplementary_Data [file fcaf432_supplementary_data.zip › Supplementary_material.pdf]

Supplementary Methods

Intermittent Access 2-Bottle Choice

Starting on postnatal day 46, rats began the IA2BC protocol. On Monday-Wednesday-Friday one 100 ml bottle contained (EtOH) 20% ethanol concentration in potable water, while the other 100 ml bottle held plain potable water (H_2O) (Figure 1b). On Tuesday-Thursday-Saturday-Sunday the two bottles had 100 ml of potable water. These days could be considered as withdrawal periods. The measures of ethanol intake were obtained on Tuesday-Thursday-Saturday in g/kg/24-hour units. The placement of the ethanol bottle was randomized in each drinking session to control for side preferences. Rat cages at different stages were randomly allocated after each procedure by JR-T, DA-V, CC-A and AL-C. Thirty minutes and twenty-four hours after placement, the EtOH bottle was weighted in order to obtain the binge, main intake, and substance preference measures (Eq. 1.):

Eq. 1:

$$Alcohol\, main\, intake = (Initial - 24hrs) \cdot Animal\, weight\, (\mathrm{g})$$

$$Binge = (Initial - 30min) \cdot Animal\, weight\, (\mathrm{g})$$

$$Preferency = Alcohol\, main\, intake \div Total\, fluid\, intake$$

Blood corticosterone concentration

For this procedure, rats were immobilized using movement restriction tubes during the extraction process, which lasted no longer than 5 minutes to prevent any acute stress response caused by handling [1,2]. Once the blood samples were collected, they were placed on ice and immediately centrifuged at 5000 rpm at 4 C for 20 min (Eppendorf centrifuge, Model 5415R). To analyze serum corticosterone concentrations, we followed the instructions provided by the commercial enzyme-linked immunosorbent assay (ELISA) kit for corticosterone (ALPCO, Cat. 55-CORMS-E01). Using a spectrophotometer (Varioskan LUX Multimode Microplate Reader v.4.00; Thermo Fisher Scientific, MA, USA) absorbance was measured at 450 nm, and serum concentration (ng/ml) was determined through the four-parameter logistic equation (4-PL).

Behavioral tests

The EPM test apparatus consisted of a plus-shaped maze with two open arms (45 cm × 10 cm each) and two enclosed arms (45 cm × 10 cm each), connected by a central platform (10 cm × 10 cm). The maze was elevated 40 cm above the floor, with illumination above 450 lux under open arms and 0-50 on closed arms as recommended previously [3]. Individual rats were evaluated and recorded for five minutes (HD camera model S4612). Each video was compressed and cropped to the dimensions of the maze for subsequent software analysis by using ffmpeg v.4.2.7.

DeepLabCut training network for EPM was created using 20 distinct frames randomly selected from each of 14 videos of different animals. Each frame was manually labeled in a top-down view for each segment of the maze (top, down, left, right) and eleven body parts of the rats (nose, head center, neck, body center, body center left, hip joint left, hip joint right, tail base, tail center, and tail tip). New videos were continually added, requiring re-labeling and re-training of the network. A ResNet-50 based neural network with default parameters was used for training with a total of 2,030,000 iterations. The trained network achieved a test error of 2.17 pixels and a training error of

2.64 pixels. With a p-cutoff value of 0.6, the training error improved to 2.45 pixels, and the test error decreased to 1.9 pixels. Data tracking generated by DeepLabCut was further processed using DLCAnalyzer [4] in R programming language (see Code Availability section for details). The distance traveled, velocity, number of entries into each zone (open arms, closed arms, and center platform), and time spent in each zone were based on the data tracking. The time spent and number of entries in each arm were used to calculate an anxiety index (Eq. 2). A higher anxiety index value corresponds to greater anxiety-like behavior in the rat.

Eq. 2:

$$Anxiety\ index = 1 - \left(\left[\frac{Open\ arms\ time}{Total\ time}\right] + \left[\frac{Open\ arm\ entries}{Total\ entries}\right] \div 2 \right)$$

The novel object recognition (NOR) test assessed rat recognition memory and recall of the memory process [5] by measuring the innate exploratory behavior of a novel object in comparison to a familiar one [6]. The test was conducted in a square arena (40 cm × 40 cm) following a three-stage protocol: 1) habituation to the polycarbonate box with wood chips bedding for 10 minutes (40 cm square arena), 2) familiarization: for 3 minutes it was allowed to the rats to get familiarized with two identical glass jars (transparent glass and metal lid) placed in the center of the arena, separated by ~10 cm, and 3) recognition test: one familiar object was replaced with a novel object having distinct physical characteristics (transparent glass light bulb with a metal cap). Then, rats were allowed to explore the arena for 3 minutes. The locations of familiar and novel objects were counterbalanced across trials (left/right). All objects were cleaned with 70% ethanol between each phase. Individual rats were video recorded with an HD camera (model S4612) during the familiarization and recognition test phases.

Videos were preprocessed using ffmpeg to ensure consistent dimensions and object positions. Similar to the EPM test, automated behavioral tracking was performed using DeepLabCut v. 2.3.8. Two separate neural networks were trained, one for each test phase (familiarization and recognition). Each network was trained using 10 distinct frames selected from 10 videos of different animals per phase. New videos were continually added, necessitating re-labeling and re-training. As with the EPM analysis, a ResNet-50 based neural network with default parameters was used for training until a total of 1,620,000 iterations was reached. The recognition trained network got a test error of 3 pixels and a train error of 3 pixels. With a p-cutoff of 0.6, a train error of 2.74 pixels, and a test error of 3.06 pixels. Data tracking generated by DeepLabCut was further processed using a custom DLCAnalyzer script. Time spent exploring familiar and novel objects was used to calculate the discrimination ratio (Eq. 3).

Eq. 3:

$$Discrimination\ ratio = \frac{Recognition\ time\ to\ novel\ object\ (seconds)}{Recognition\ time\ to\ novel\ object\ (s) + Recognition\ time\ to\ familiar\ object\ (s)}$$

The conditioned place preference (CPP) is a preclinical modeling paradigm aimed at studying the rewarding and aversive effects of substances like ethanol [7,8]. The apparatus consisted of a three-chambered box with distinct visual and tactile features (white walls/rough floor with ethanol access, gray walls/smooth floor, black walls/smooth floor with water access). The test evaluated the animal's compartment preference across three stages: 1) pre-test (baseline time spent in each compartment without any substance), 2) habituation (6 days to eliminate novelty and for

chamber-stimulus association), and 3) test (evaluation of compartment time spent). The preference index was calculated using the time (in seconds) in each chamber (Eq. 4).

Eq. 4:

$$Preference\ index\ =\ \frac{EtOH\ chamber\ time}{Total\ time\ on\ EtOH\ and\ H2O\ time}$$

Supplementary figure 1. Labels used to train the deeplabcut networks.

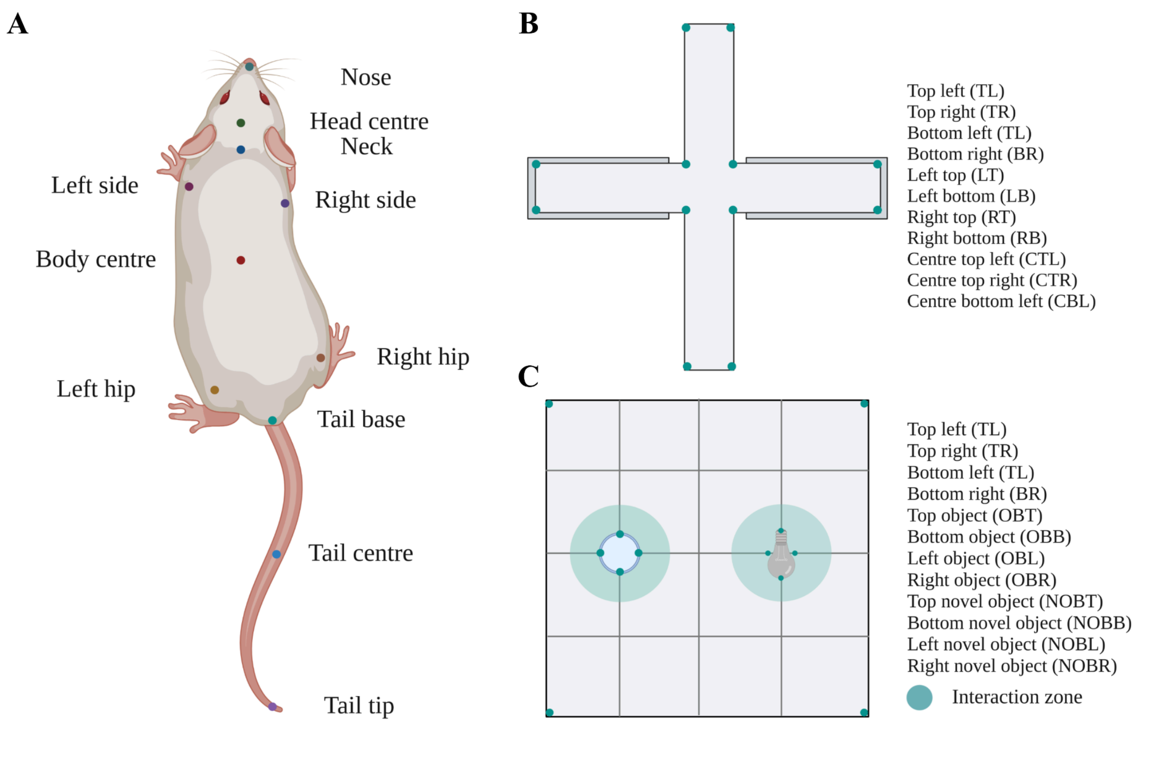

A) The points of interest used to track the rat in each maze, B) The points of interest in the novel object recognition test, C) The points of interest in the elevated plus maze.

Magnetic Resonance Imaging

Structural MRI (sMRI) was acquired with a 3D FLASH sequence T2w with 2 repetitions, TR=30.76 ms, TE=5 ms, flip angle=10º, FOV=25.6x19.098x25.6 mm, and an isometric voxel of 160 microns. For the resting state functional MRI (rsfMRI), we acquired a Gradient-echo Echo-planar imaging (GE-EPI) sequence with the following parameters TR=1,000 ms, TE=20 ms, flip angle=60º, slice thickness=1mm, FOV=30x30, number of slices=24, volumes=600. The interface software used was Paravision v.7.0.

Structural analysis

All MRI scans were converted from Bruker format to NIfTI using the *brkraw* toolbox v0.3.11[9] using the BIDS framework[10]. We then preprocessed the T2w images with intensity normalization[11], centering of the image, and denoise by using an in-house pipeline built on MINC-toolkit-v2 and ANTs tools and based on previous studies.[12] One T2w image (1 subject-1 session) was excluded due to the high level of artifacts during the quality control stage. Finally, we performed Deformation Based Morphometry (DBM) employing a Two-Level DBM approach with the SIGMA anatomical template v.1.2.1.[13] We analyzed the voxel-wise longitudinal volume changes to find possible interactions between group, age, and sex. We used relative volume (Jacobian determinant relative to the averaged template created) per voxel as the dependent variable and age, group, and sex as independent variables.

Finally, to analyze the relationship between MRI brain volume and behavioral outcomes, we employed partial least squares (PLS) correlation.[14] This multivariate approach allowed us to identify distributed patterns in brain structure related to behavior. For this analysis we associated the MRI-T3 with Behav-T2 acquisition points as they were acquired in adjacent time points.

Functional analysis

For the resting state functional MRI (rsfMRI) data preprocessing, we employed the open-source RABIES pipeline for multi-stage correction.[15] To identify subnetworks (connected components) in rsfMRI that differ significantly by alcohol and stress conditions while controlling for multiple comparisons across a large number of connections, we used the R-library network-based R-statistics (NBR) that allows to perform network based statistics (NBS) with linear mixed-models.[16,17] We identified a connected subnetwork altered by the CRS (Figure 5a), and then, as a post hoc analysis, we focused on how the functional connectivity (FC) trajectory changed for each connection (ROI-to-ROI) within this subnetwork, filtering the most relevant connections by selecting the connections with significant group differences. Again, we used linear mixed-models with group, age, and sex interactions, batch as the covariate, and subject ID as a random effect (Eq. 1).

Statistical analysis

We evaluated the effect of chronic stress and/or ethanol intake with relative volume, functional correlation, weight change, ethanol intake, anxiety index, and preference index as dependent variables in different models (Eq. 5). Group, session, and sex were included as interaction fixed effects (independent variables). In contrast, the discrimination ratio was analyzed using a linear regression model for the NOR task (Eq. 6).

To assess group differences in MRI analyses, linear mixed-effects models were applied (Eq. 7). Batch served as a covariate in all models, while group, age, and sex were included as interaction fixed effects.

Eq. 5:

$$Metric \sim Group \bullet Age \bullet Sex + Group + Age + Sex + Batch \; + (1|RID)$$

Metrics: local volume, functional connectivity, ethanol main intake, Δweight = (weight in each session - weight of the first session), anxiety index, and preference index.

Eq. 6:

$$Metric \sim Group \bullet Sex + Group + Sex + Batch$$

Metrics: discrimination ratio.

Statistical assumptions for each model were implemented in R-language v.4.1 using the easystats and performance libraries (See code availability section)

References

1. Deacon RMJ. Housing, husbandry and handling of rodents for behavioral experiments. *Nat Protoc*. 2006;1(2):936-946. doi:10.1038/nprot.2006.120

2. Benedetti M, Merino R, Kusuda R, et al. Plasma corticosterone levels in mouse models of pain. *Eur J Pain*. 2012;16(6):803-815. doi:10.1002/j.1532-2149.2011.00066.x

3. Neuwirth LS, Verrengia MT, Harikinish-Murrary ZI, Orens JE, Lopez OE. Under or absent reporting of Light stimuli in testing of anxiety-like behaviors in rodents: The need for standardization. *Front Mol Neurosci*. 2022;15:912146. doi:10.3389/fnmol.2022.912146

4. Sturman O, von Ziegler L, Schläppi C, et al. Deep learning-based behavioral analysis reaches human accuracy and is capable of outperforming commercial solutions. *Neuropsychopharmacology*. 2020;45(11):1942-1952. doi:10.1038/s41386-020-0776-y

5. Antunes M, Biala G. The novel object recognition memory: neurobiology, test procedure, and its modifications. *Cogn Process*. 2012;13(2):93-110. doi:10.1007/s10339-011-0430-z

6. Ennaceur A, Delacour J. A new one-trial test for neurobiological studies of memory in rats. 1: Behavioral data. *Behav Brain Res*. 1988;31(1):47-59. doi:10.1016/0166-4328(88)90157-x

7. Tzschentke TM. Measuring reward with the conditioned place preference paradigm: a comprehensive review of drug effects, recent progress and new issues. *Prog Neurobiol*. 1998;56(6):613-672. doi:10.1016/s0301-0082(98)00060-4

8. Prus AJ, James JR, Rosecrans JA. Conditioned Place Preference. In: Buccafusco JJ, ed. *Methods of Behavior Analysis in Neuroscience*. CRC Press/Taylor & Francis. https://www.ncbi.nlm.nih.gov/pubmed/21204336

9. Lee SH, Ban W, Shih YYI. *BrkRaw/bruker: BrkRaw v0.3.4*. Zenodo; 2020. doi:10.5281/ZENODO.3907018

10. Gorgolewski KJ, Auer T, Calhoun VD, et al. The brain imaging data structure, a format for organizing and describing outputs of neuroimaging experiments. *Sci Data*. 2016;3:160044. doi:10.1038/sdata.2016.44

11. Tustison NJ, Avants BB, Cook PA, et al. N4ITK: improved N3 bias correction. *IEEE Trans Med Imaging*. 2010;29(6):1310-1320. doi:10.1109/TMI.2010.2046908

12. Lerch JP, Yiu AP, Martinez-Canabal A, et al. Maze training in mice induces MRI-detectable brain shape changes specific to the type of learning. *Neuroimage*. 2011;54(3):2086-2095. doi:10.1016/j.neuroimage.2010.09.086

13. Barrière DA, Magalhães R, Novais A, et al. The SIGMA rat brain templates and atlases for multimodal MRI data analysis and visualization. *Nat Commun*. 2019;10(1):1-13. doi:10.1038/s41467-019-13575-7

14. McIntosh AR, Lobaugh NJ. Partial least squares analysis of neuroimaging data: applications and advances. *Neuroimage*. 2004;23 Suppl 1:S250-S263. doi:10.1016/j.neuroimage.2004.07.020

15. Desrosiers-Grégoire G, Devenyi GA, Grandjean J, Chakravarty MM. A standardized image processing and data quality platform for rodent fMRI. *Nat Commun*. 2024;15(1):6708. doi:10.1038/s41467-024-50826-8

16. Gracia-Tabuenca Z, Alcauter S. NBR: Network-based R-statistics for (unbalanced) longitudinal

samples. *bioRxiv*. Published online November 8, 2020:2020.11.07.373019. doi:10.1101/2020.11.07.373019

17. Zalesky A, Fornito A, Bullmore ET. Network-based statistic: identifying differences in brain networks. *Neuroimage*. 2010;53(4):1197-1207. doi:10.1016/j.neuroimage.2010.06.041

Supplementary results

Behavioral test

Individual EPM metric analysis showed that ethanol female groups (EtOH+/CRS- and EtOH+/CRS+), compared to the female EtOH-/CRS- group, spent less percentage time moving (q = 0.006, q = 0.004) with less speed (q = 0.031, q = 0.022), but more stationary time (q = 0.02, q = 0.013) and total spent time (q = 0.04, q = 0.029) in the closed top arm. CRS male groups (EtOH-/CRS+ and EtOH+/CRS+) only showed significant results compared with the male EtOH-/CRS- group. The EtOH-/CRS+ males spent more time moving (q = 0.037), specifically in the center (q = 0.015), and spent more time (q = 0.044) in the closed bottom arm. Compared with male control group, the male EtOH+/CRS+ group moved during more time (q = 0.012) with more distance covered (q = 0.009), more transitions into (q = 0.04), and spending more time (q = 0.048) in the center of the arm, but more stationary time (q = 0.048) in both closed arms. Less transitions (q = 0.027) to the closed top arm, more distance covered (q = 0.034), more transitions into (q = 0.014), time (q = 0.03) and distance moving (q = 0.03) in the left open arm, and less transitions (q = 0.025) in the open right. This group also showed more transitions to the left (q = 0.025, q = 0.002) and right arm (q = 0.009, q = 0.029).

Discrimination ratio of the NOR task (Eq. 3, supplementary results) showed a lower index only in the male EtOH-/CRS+ group compared with male EtOH+/CRS- (β = 0.26, q = 0.023, p = 0.003, d = 1.53), and uncorrected significant compared with male EtOH-/CRS- (β = 0.19, q = 0.096, p = 0.048, d = 1.1) and with male EtOH+/CRS+ (β = -0.17, q = 0.096, p = 0.048, d = -0.97) (Figure 3d). In particular, this group spent less time in the novel object (q = 0.044) with less stationary time (q = 0.037) and speed moving (q = 0.046). Interestingly, the male EtOH+/CRS+ group moved more distance (q = 0.026) with higher speed (q < 0.001) compared with male EtOH+/CRS-, into the familiar object (speed moving: q = 0.012), in the novel one (speed moving: q = 0.028), and showed higher speed compared with EtOH-/CRS- in familiar (q = 0.019) and novel (q = 0.039) objects. In contrast, females EtOH-/CRS+, compared with EtOH-/CRS- of the same sex, showed higher time spent (q = 0.025), distance moved (q = 0.04) and moving time (q = 0.011) in the familiar object. In the similar way that males, the EtOH+/CRS+ female group showed more distance moving (q = 0.025) compared with EtOH+/CRS-, into the familiar object (speed moving: q = 0.04) and in the novel one (speed moving: q = 0.015). Besides, this contrast also showed spending more stationary time (q = 0.04) in both object transitions (familiar: q = 0.035, novel: q = 0.025) and distance moving (familiar q = 0.015, novel: q = 0.015). Besides, female EtOH+/CRS+ contrasted to EtOH-/CRS- group had more distance moving (q = 0.004), total time spent (q = 0.022) and transitions (q = 0.001) in familiar object, but more distance moving (q = 0.015) and time moving (q = 0.048) in only novel object.

Voxelwise analysis of each group contrasted with the control

Ethanol intake groups (EtOH+/CRS- and EtOH+/CRS+) exhibited an enlargement in the entorhinal cortex (Ent) and thalamus (Thal), along with a reduction in the hypothalamus (Hyp) and cerebellum. Conversely, chronic restraint groups (EtOH-/CRS+ and EtOH+/CRS+) showed an increase solely in the insular cortex (Ins) and a decrease in the thalamus, secondary motor cortex (M2), and retrosplenial cortex (RSG). The EtOH+/CRS- group alone showed volume alterations in the substantia nigra (decrease) and perirhinal cortex (increase). The EtOH-/CRS+ group displayed an increase in the orbitofrontal lobule (Orb), while EtOH-/CRS+ exhibited an expansion in the secondary cingulate cortex (Cg2) and a reduction in the secondary motor cortex (M2). The amygdala (Amy) region showed opposite volume changes between EtOH+/CRS- (higher) and EtOH-/CRS+ (lower) compared to the control group (See supplementary results).

Compared to the only stress (EtOH-/CRS+) group, ethanol intake groups exhibited increased volumes in the olfactory bulb (OB), cerebellum (Cer), entorhinal cortex (MEntR), and thalamus (Thal), alongside decreased volumes in the dorsal caudate-putamen (CPu) and Thal. Additional regions, such as the nucleus accumbens (Nacc), hippocampus (Hipp), insular cortex (Ins), and hypothalamus (Hyp), showed increased volumes in the EtOH+/CRS- group but decreased volumes in the EtOH+/CRS+ group. The substantia nigra (SN) and secondary motor cortex (M2) displayed increased volumes, whereas the frontal cortex, secondary cingulate cortex (Cg2), and amygdala (Amy) exhibited decreased volumes. The orbitofrontal cortex (Orb), retrosplenial cortex (RSG), and primary motor cortex (M1) showed reduced volumes exclusively in the EtOH+/CRS+ group. In contrast to ethanol intake (EtOH+/CRS-), chronic restraint stress led to increased volumes in the OB, Cer, Cg2, MEntR, CPu, and Thal in both groups (EtOH+/CRS+ and EtOH-/CRS+). This increase was accompanied by a decrease in the volumes of the Hipp (DG), Thal, and Amy (See supplementary results).

Longitudinal functional connectivity analysis

The female EtOH+/CRS- rats exhibited altered connectivity between the right amygdala and the left vermis of the cerebellum (rAmy-lvCer, figure 6b), while the female EtOH-/CRS+ group showed altered connectivity between the right amygdala and the left thalamus (rAmy-lThal, figure 6c). The female EtOH+/CRS+ group showed altered significant connectivity vs the control in the connection of the right secondary cingulate cortex with the left secondary motor cortex (rCg2-lM2, figure 6d). The three experimental groups displayed altered connectivity between the right secondary cingulate cortex and the right thalamus (rCg2-rThal, figure 6e), but only the EtOH+/CRS+ group had altered connectivity of right Cg2 with the left secondary motor cortex. Additionally, both female ethanol groups showed altered connectivity between the right hippocampus and the right vermis of the cerebellum (rHipp-rvCer, figure 6f) contrasted with EtOH-/CRS+. On the other hand, only male EtOH+/CRS+ rats exhibited altered connectivity between the left primary cingulate cortex and the left striatum (lCg1-lStr, figure 6g) in comparison to the male EtOH+/CRS- group, as well as between the right entorhinal cortex and the left substantia nigra (rEnt-lSN, figure 6h) in contrast to the male EtOH-/CRS+ group.

Supplementary tables

Supplementary table 3. Group pairwise comparison of longitudinal ROI functional connectivity changes based on estimated marginal means.

Contrast		ROI	ROI	Sex	Mean	d	SE	df	p-value	q-value
EtOH-/CRS-	EtOH+/CRS-	rAmy	lvCer	fem	0.08	0.82	0.02	77	< 0.001	0.01
		rCg2	rThal	fem	-0.09	-0.83	0.03	77	< 0.001	< 0.001
	EtOH-/CRS+	rAmy	lThal	fem	0.1	0.86	0.04	80	0.01	0.04
		rCg2	rThal	fem	-0.12	-1.04	0.03	80	< 0.001	< 0.001
	EtOH+/CRS+	rCg2	lM2	fem	-0.11	-0.64	0.04	82	0.01	0.049
		rCg2	rThal	fem	-0.15	-1.04	0.03	81	< 0.001	< 0.001
EtOH+/CRS-	EtOH-/CRS+	rHipp	rvCer	fem	0.08	0.77	0.03	78	0.01	0.04
	EtOH+/CRS+	lCg1	lStr	male	0.1	-0.21	0.03	81	0.01	0.03
EtOH-/CRS+	EtOH+/CRS+	rHipp	rvCer	fem	-0.09	0.69	0.03	74	< 0.001	0.02
		rEnt	lSN	male	-0.08	-0.56	0.03	76	< 0.001	0.03

Abbreviation: Mean = estimated marginal means, d = effect size, SE = standard error, df = degree of freedom, Q-value = p-value fdr adjusted.

Supplementary figures

**Supplementary figure 2**. Ethanol intake between females and males in the EtOH+/CRS- group.
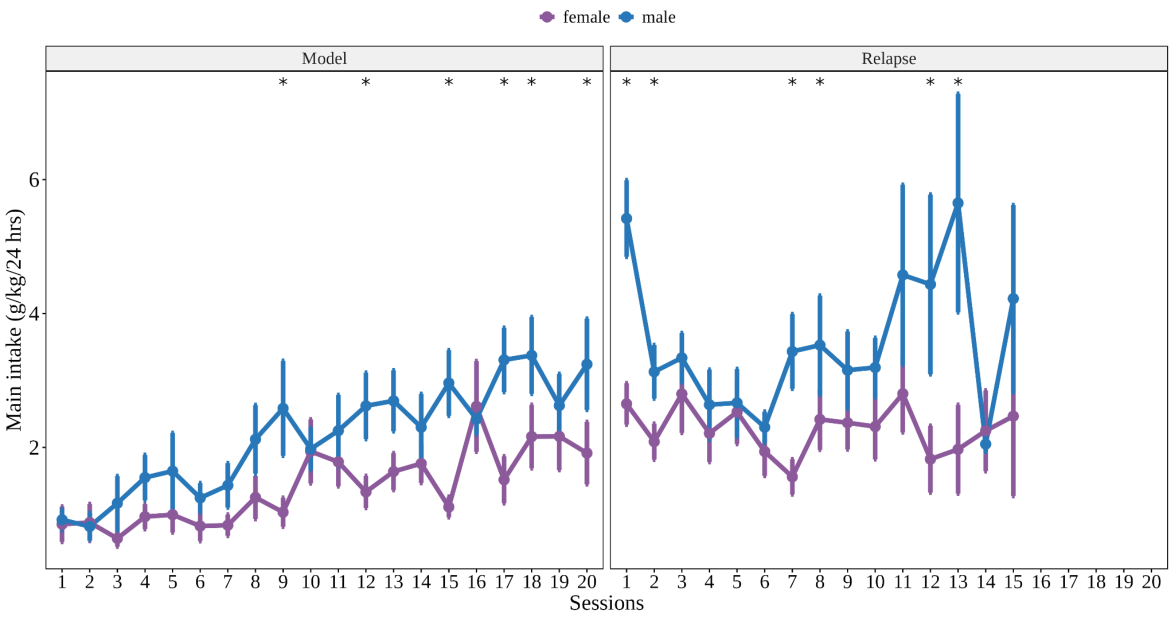
Males consumed higher levels of ethanol compared to females in the EtOH+/CRS- group. Mean intake and standard error for (n=28 data points per session for both EtOH+ groups, extreme values were excluded). Experimental unit: individual ethanol intake with rat-ID as a random effect. Group differences were assessed by a linear mixed model, formula: ethanol/binge intake ~ Sex·Age+Batch+(1|RID). Extreme values (above Q3 + 3xIQR or below Q1 - 3xIQR) were removed based on the interquartile range (IQR). Estimates, confidence intervals and associated q-values can be found in supplementary table 4.

Supplementary figure 3. Ethanol intake between females and males in the EtOH+/CRS+ group

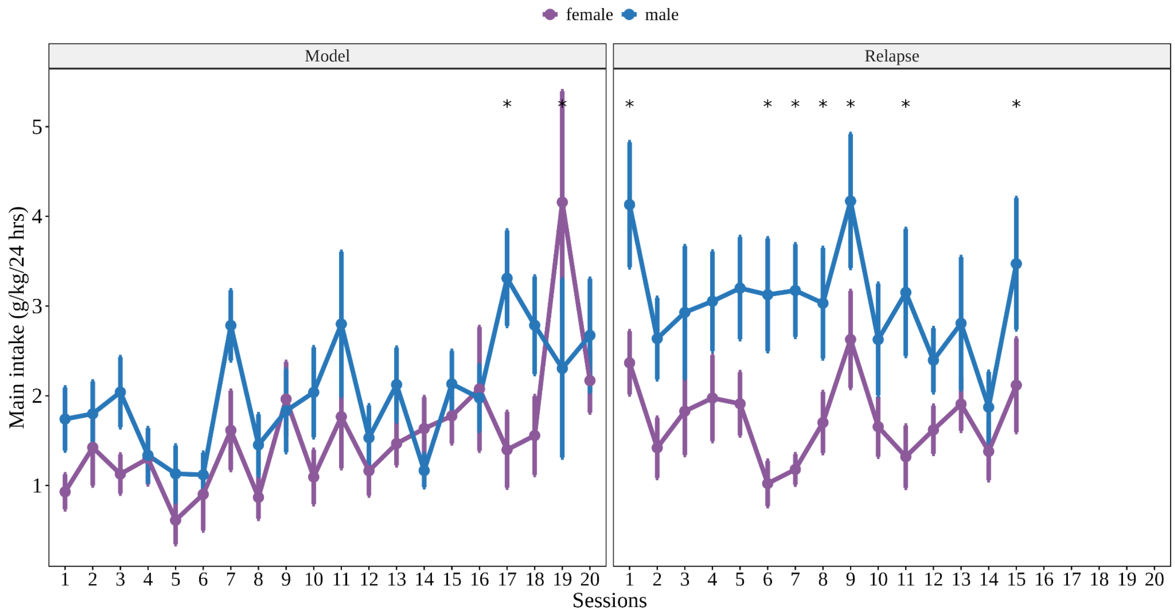

Males consumed higher levels of ethanol compared to females in the EtOH+/CRS+ group. Mean intake and standard error for (n=28 data points per session for both EtOH+ groups, extreme values were excluded). Experimental unit: individual ethanol intake with rat-ID as a random effect. Group differences were assessed by a linear mixed model, formula: ethanol/binge intake ~ Sex·Age+Batch+(1|RID). Extreme values (above Q3 + 3xIQR or below Q1 - 3xIQR) were removed based on the interquartile range (IQR). Estimates, confidence intervals and associated q-values can be found in supplementary table 4.

Supplementary figure 4. Food consumption

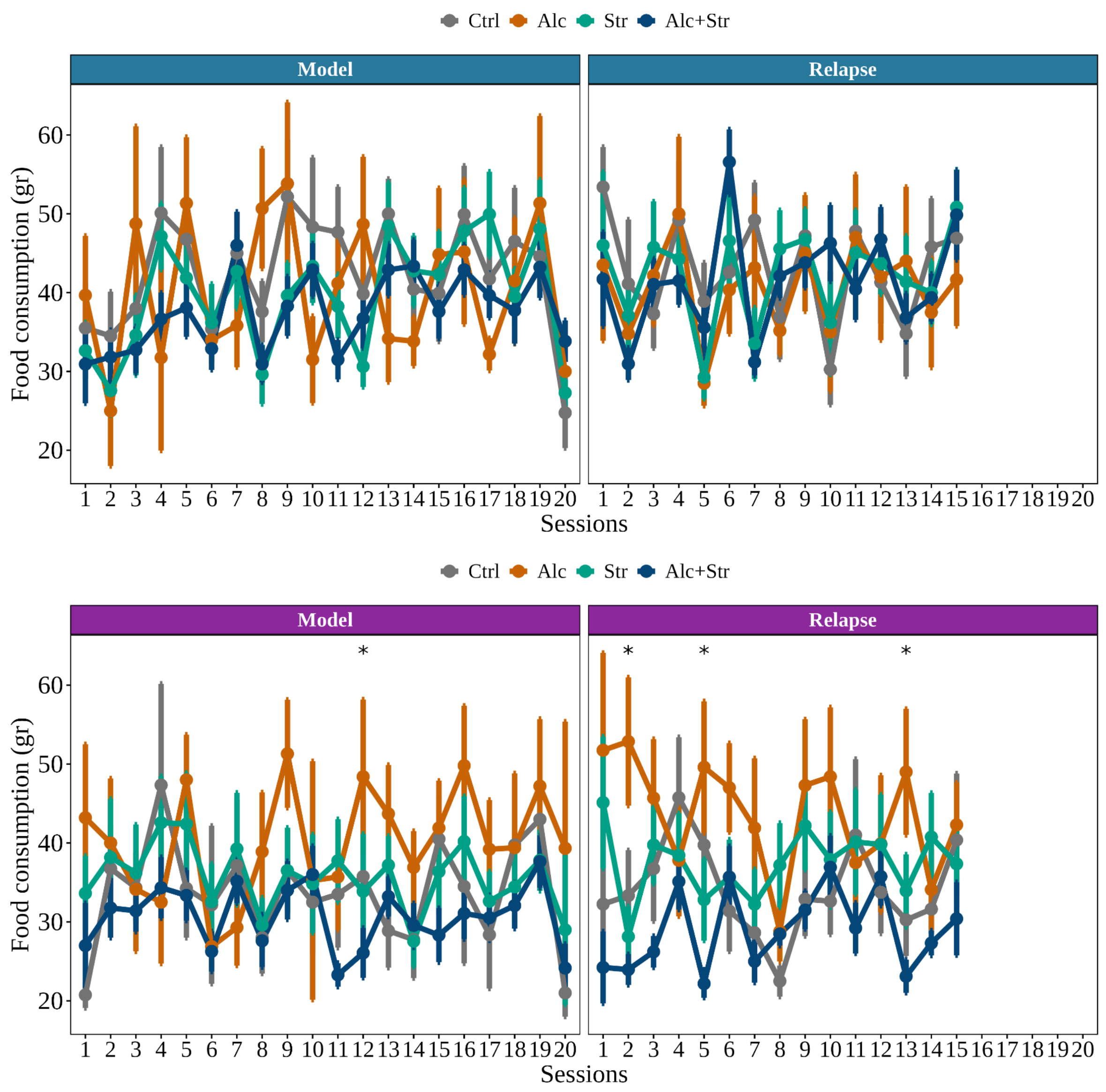

Male and female food consumption (24 hrs) over the twenty sessions of the first exposure and in the fifteen sessions of the reinstated phase. Group differences were assessed by a linear mixed model, formula: food consumption ~ Group·Sex·Age+Batch+(1|RID). Extreme values (above Q3 + 3xIQR or below Q1 - 3xIQR) were removed based on the interquartile range (IQR). Significant results were found only in females, between the EtOH+/CRS- (Alc) group with the both intervention group (Alc+Str) at sessions 12 from the first exposure model ($p = 0.047$), and sessions 2 ($p = 0.017$), 5 ($p = 0.005$) and 13 ($p = 0.011$).

Supplementary figure 5. Regional volume trajectory changes of each group contrasted to EtOH-/CRS-

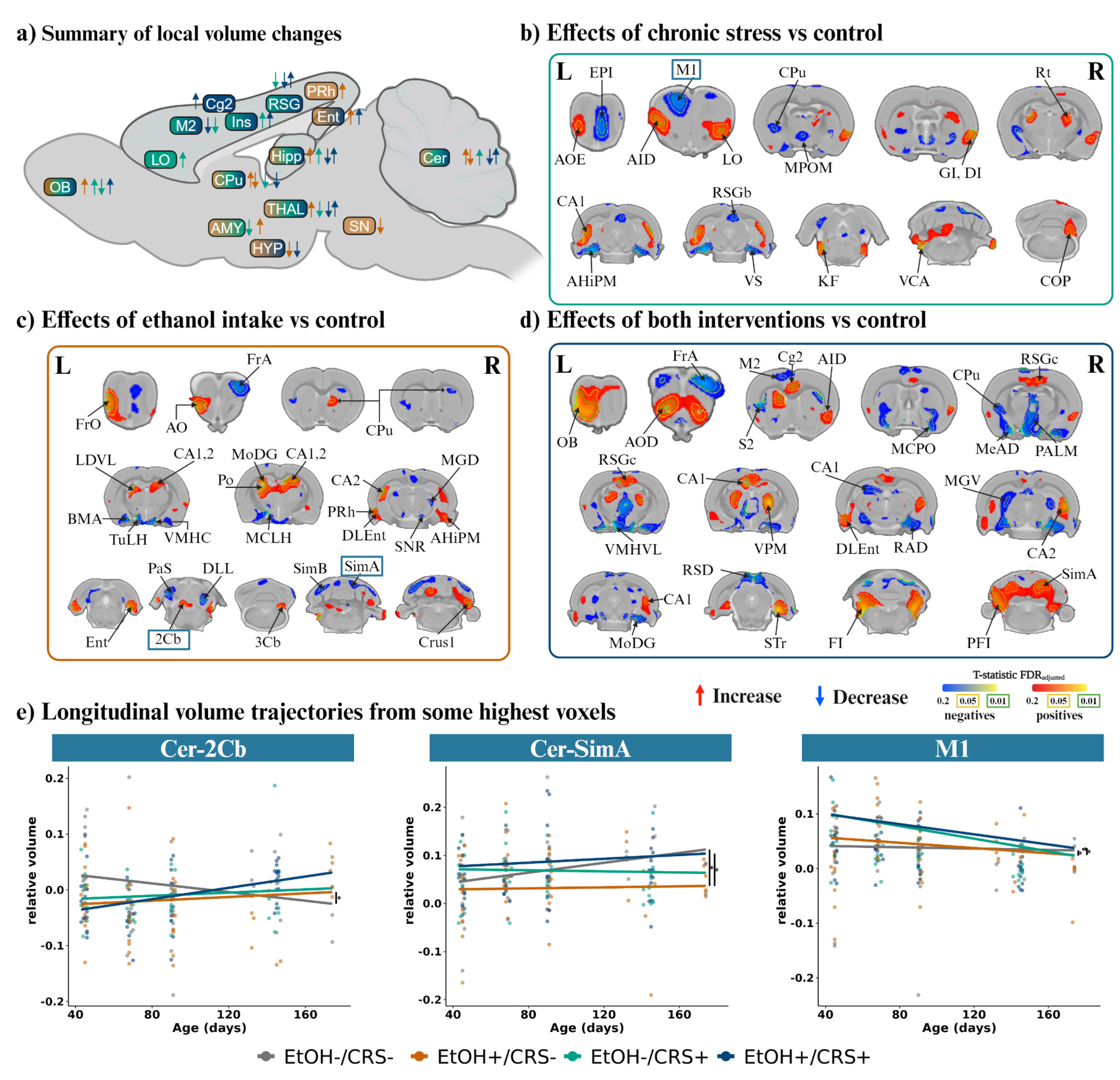

a) Schematic summary of the regional volume changes found on each contrast. **b)** Volume differences associated with stress effects (EtOH-/CRS+). **c)** Volume differences associated with ethanol effects (EtOH+/CRS-). **d)** Volume differences associated with stress and ethanol effects (EtOH+/CRS+). **e)** Scatter plot of the corrected volume with regression lines of the voxel with highest t-value of the ROI highlighted with a box (See table 3). Threshold set at FDR of 20%, 5% (yellow dashed line) and 1% (green dashed line). Coronal slides labels correspond to Paxinos & Watson stereotaxic atlas.

Supplementary figure 6. regional volume trajectory changes of each group contrasted to EtOH-/CRS+

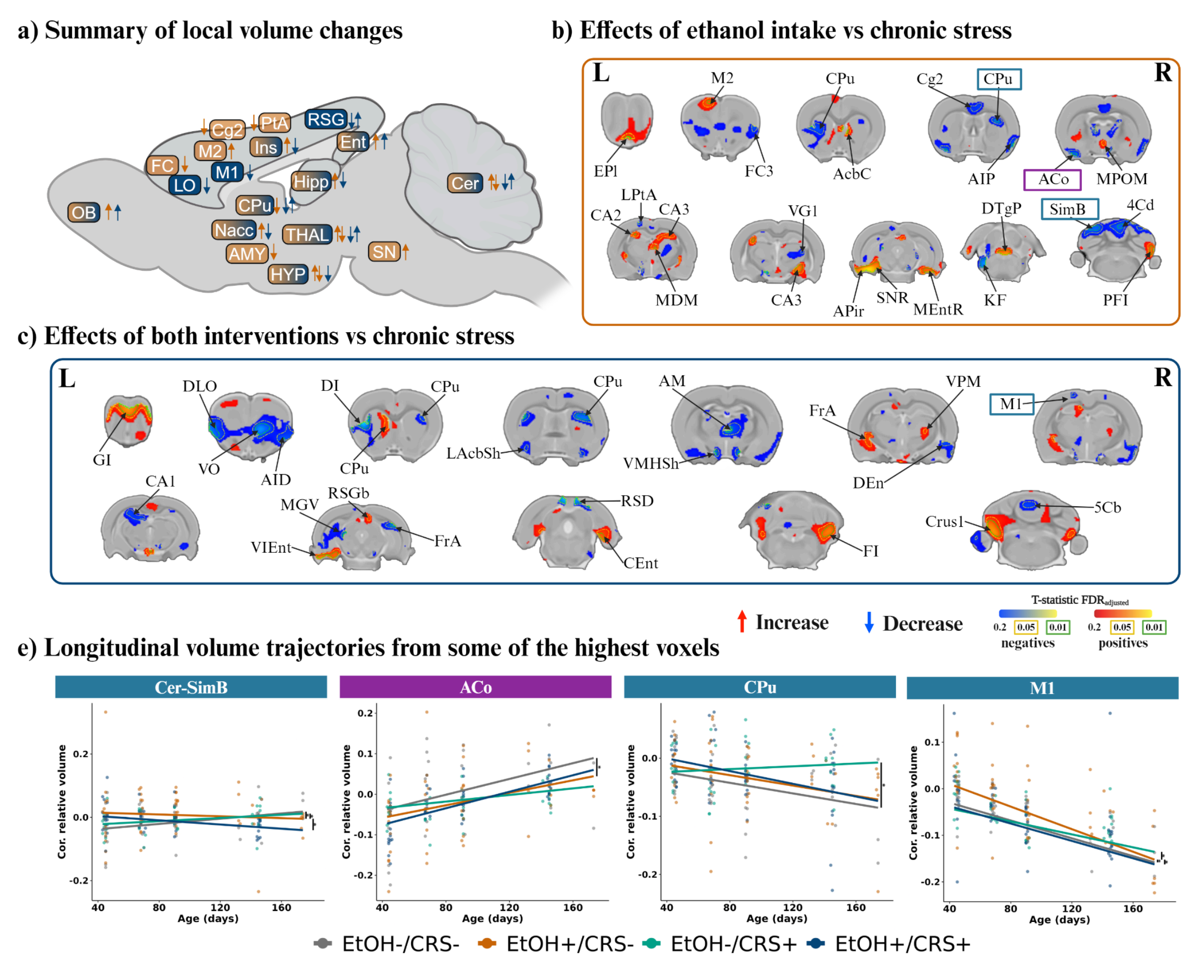

a) Schematic summary of the regional volume changes found on each contrast. **b)** Volume differences associated with ethanol effects without stress (EtOH+/CRS-). **c)** Volume differences associated with just ethanol effects (EtOH+/CRS+). **e)** Scatter plot of the corrected volume with regression lines of the voxel with highest t-value of the ROI highlighted with a box (See table 3). Threshold set at FDR of 20%, 5% (yellow dashed line) and 1% (green dashed line). Coronal slides labels correspond to Paxinos & Watson stereotaxic atlas.

Supplementary figure 7. Regional volume trajectory changes of each group compared to EtOH+/CRS-

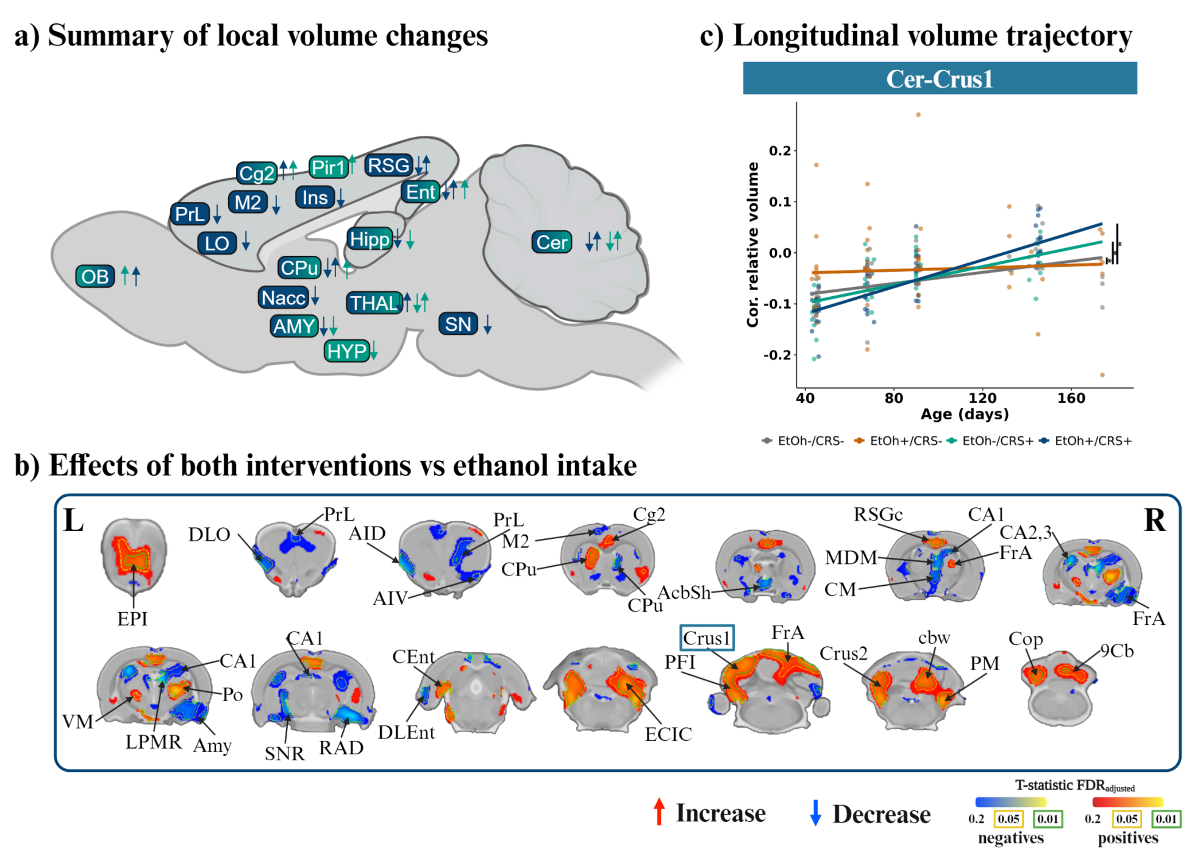

a) Schematic summary of the regional volume changes found on each contrast. **b)** Volume differences associated with just stress effects (EtOH+/CRS+). **c)** Scatter plot of the corrected volume with regression lines of the voxel with highest t-value (See table 3). Threshold set at FDR of 20%, 5% (yellow dashed line) and 1% (green dashed line). Coronal slides labels correspond to Paxinos & Watson stereotaxic atlas.

Supplementary figure 8. Corticosterone levels from a sample of each group at the end of the protocol (P142).

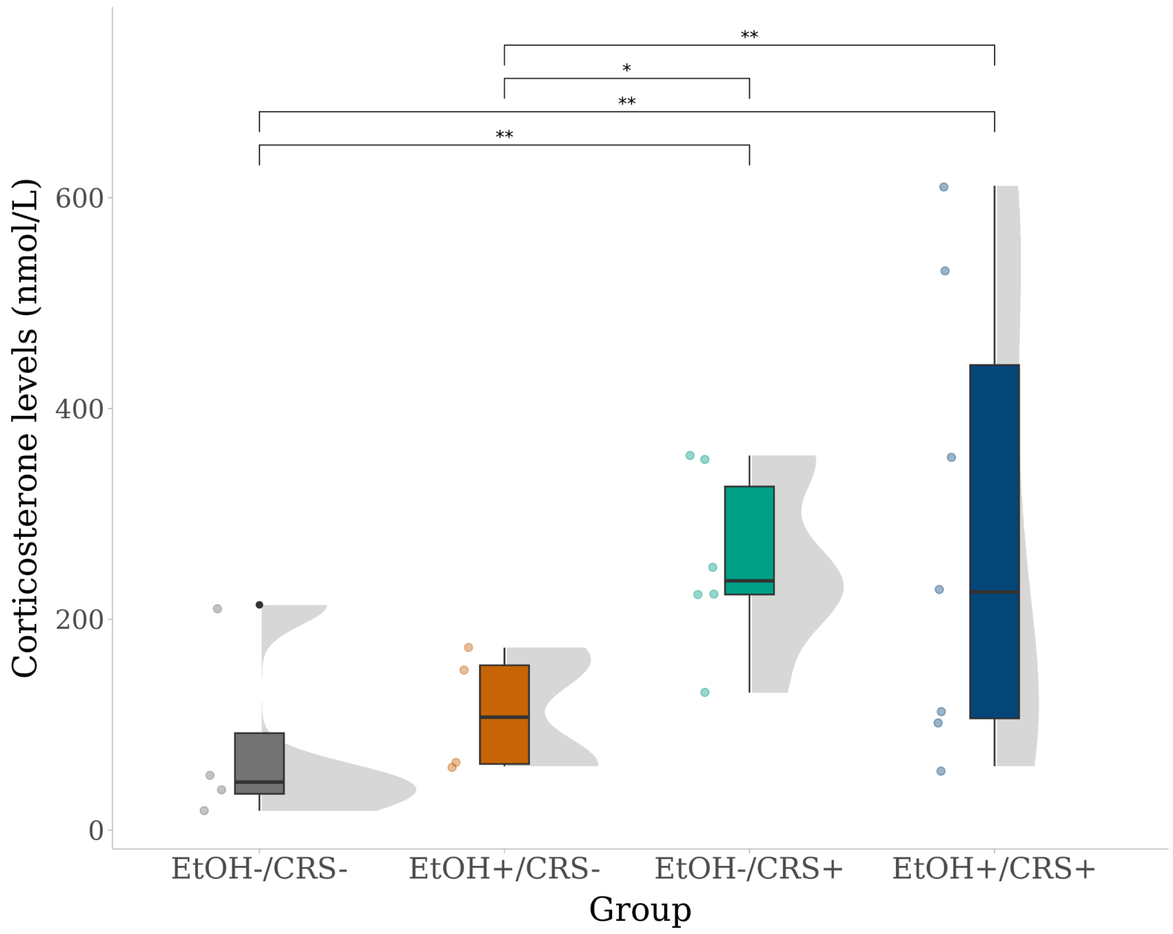

Corticosterone levels of EtOH-/CRS- (n = 4, 2 female), EtOH+/CRS- (n = 4, 2 female), EtOH-/CRS+ (n = 6, 3 female), EtOH+/CRS+ (n = 7, 3 female). Experimental unit: individual corticosterone levels with rat-ID as a random effect. Group differences were assessed by a linear mixed model, formula: corticosterone ~ Group·Age+Batch+(1|RID). Group differences: EtOH-/CRS- vs EtOH-/CRS+ (p = 0.006); EtOH-/CRS- vs EtOH+/CRS+ (p = 0.004; EtOH+/CRS- vs EtOH-/CRS+ (p = 0.013); EtOH+/CRS- vs EtOH+/CRS+ (p = 0.006).

Supplementary figure 9. Commonly reported metric comparison of elevated plus maze (EPM) and their group differences.

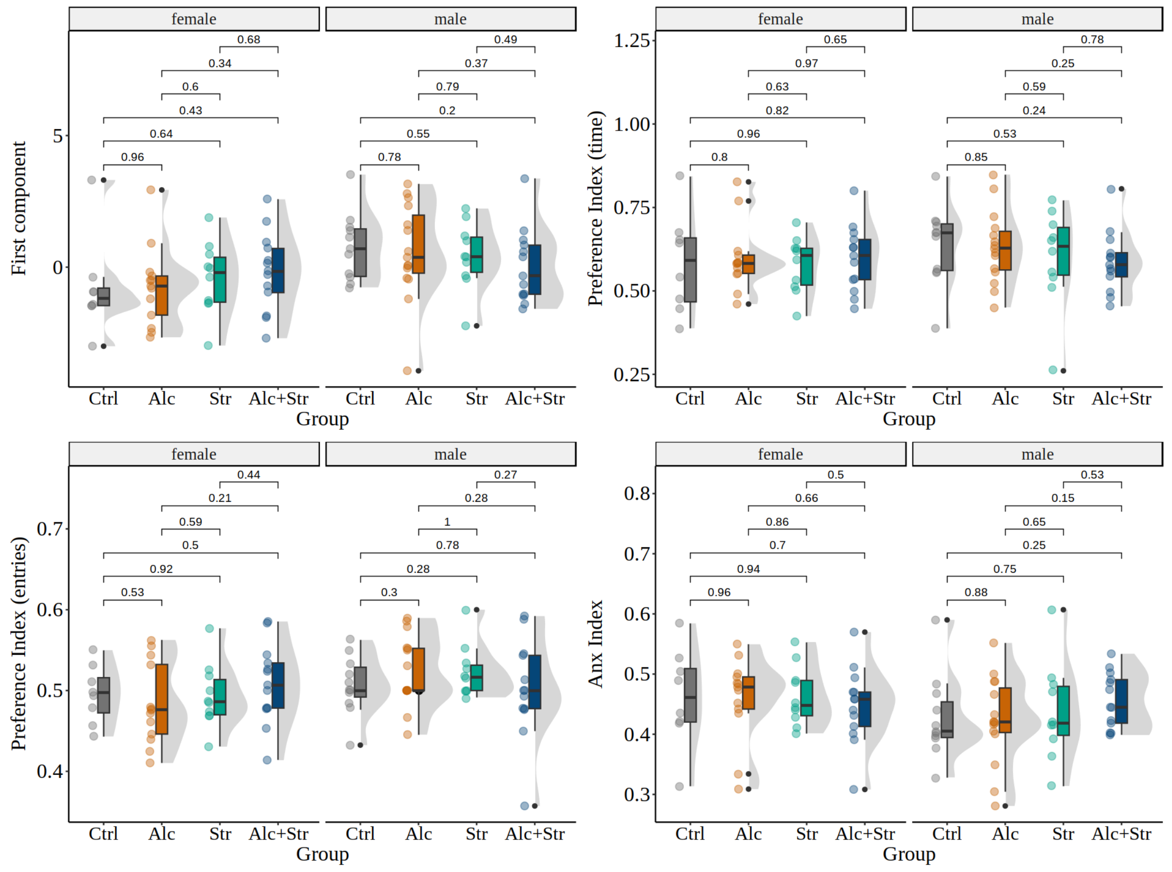

First component of a principal component analysis for all individual metrics (Supplementary figure 10-11). Preference index of time spent and entries on open arms compared with closed arms. Final anxiety index reported in the manuscript based on the average of both indexes.

Supplementary figure 10. Individual metrics of elevated plus maze (EPM) and their group differences of the females.

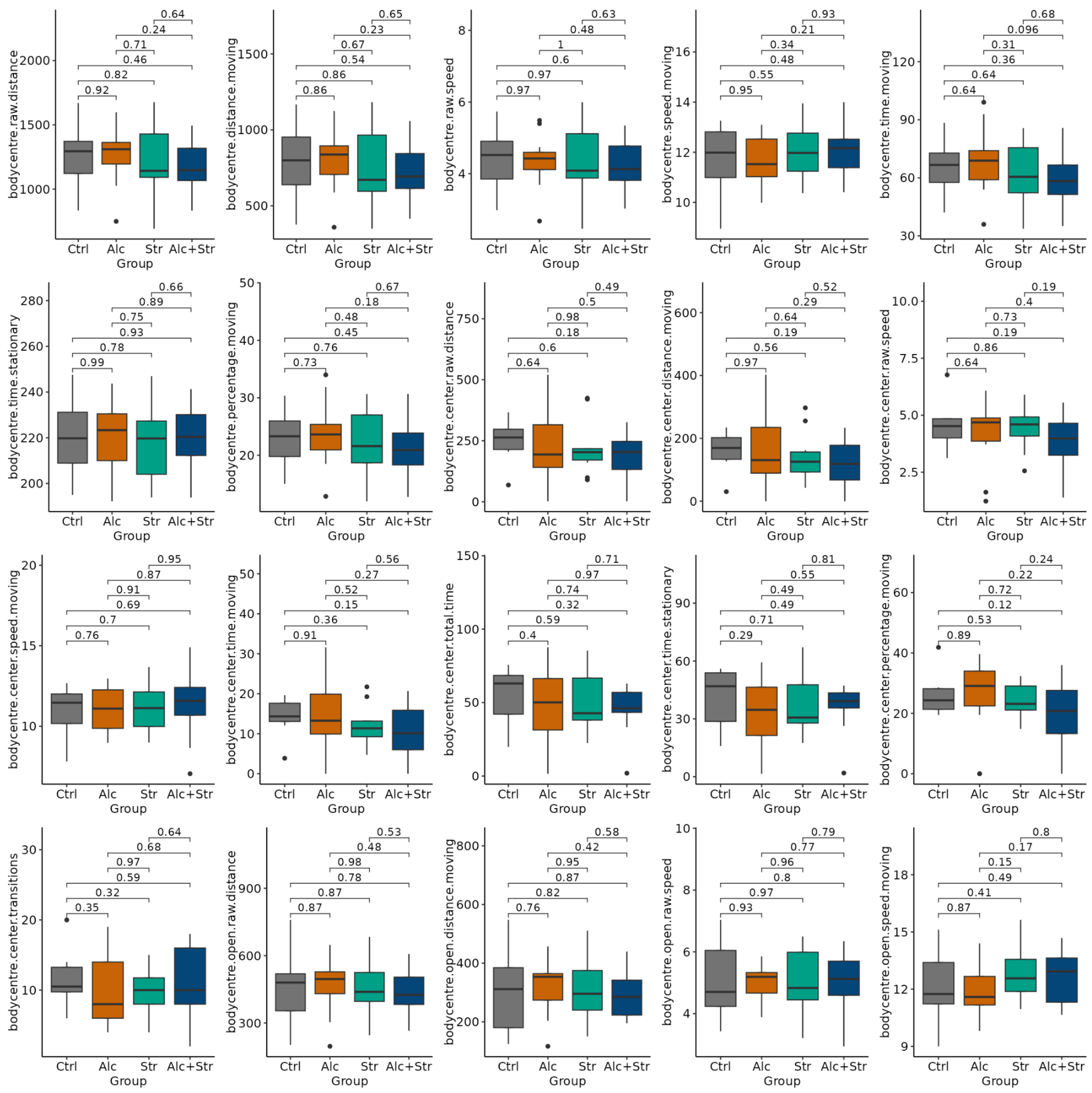

Supplementary figure 10. Individual metrics of elevated plus maze (EPM) and their group differences of the females.

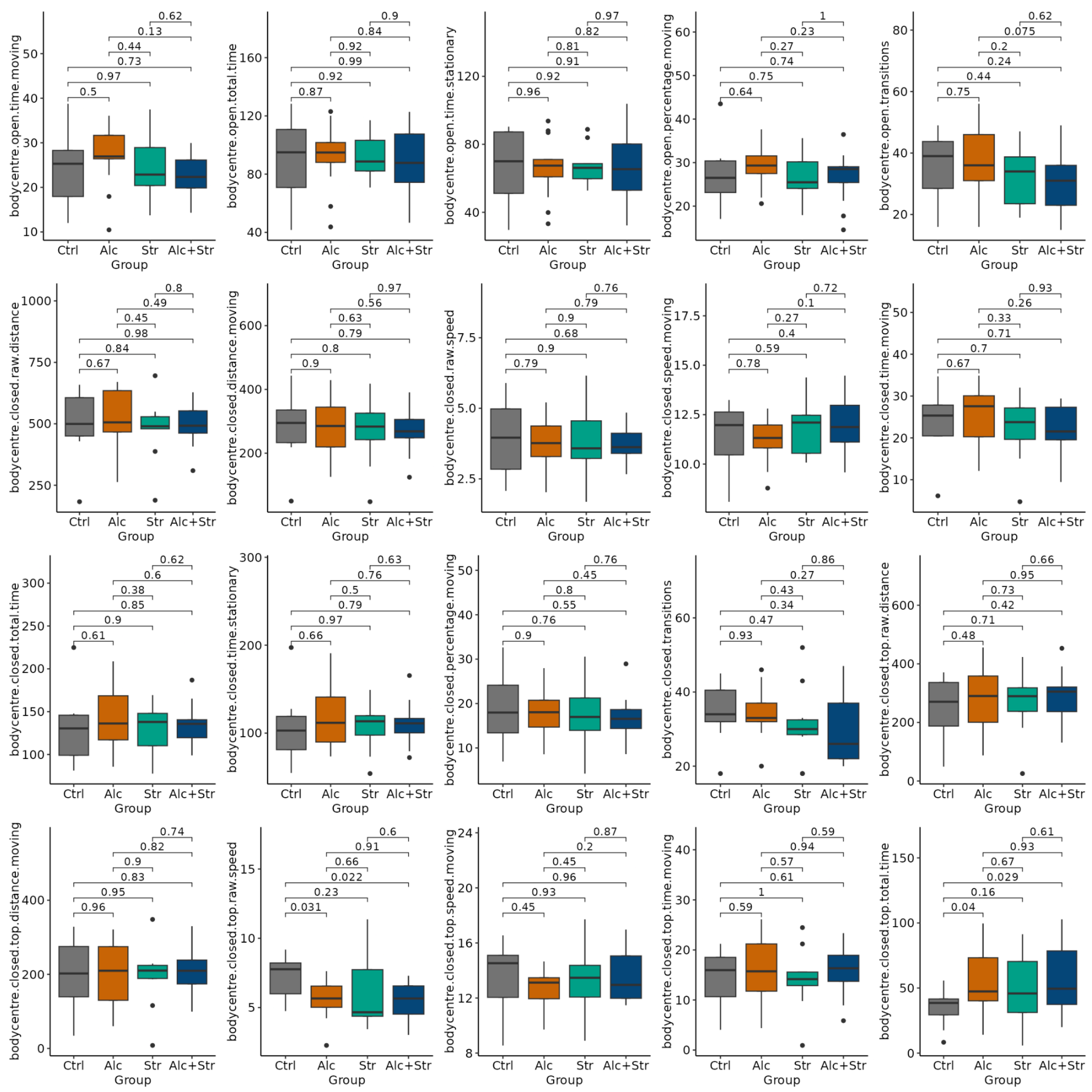

Supplementary figure 10. Individual metrics of elevated plus maze (EPM) and their group differences of the females.

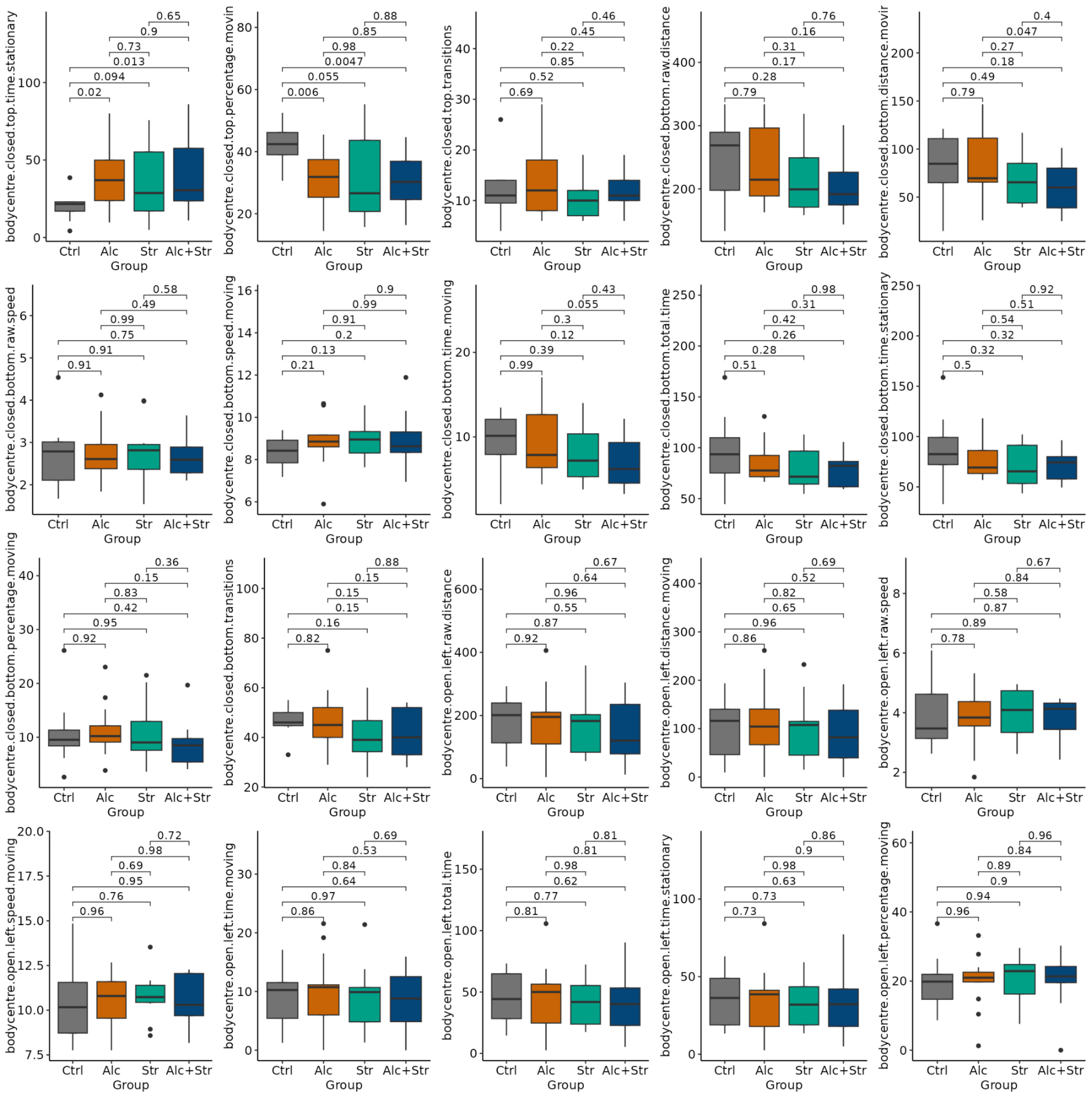

Supplementary figure 10. Individual metrics of elevated plus maze (EPM) and their group differences of the females.

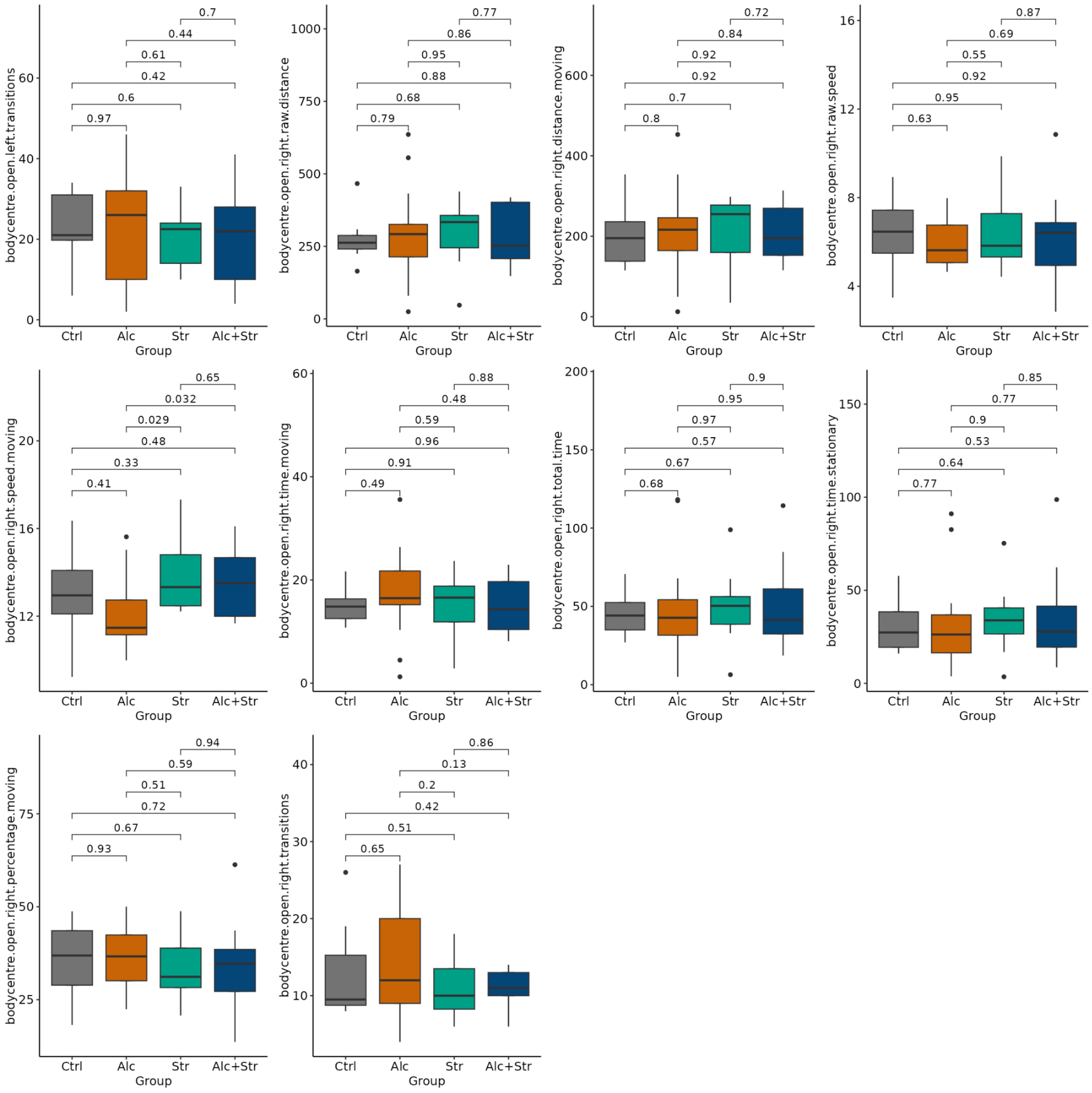

Supplementary figure 11. Individual metrics of elevated plus maze (EPM) and their group differences of the males.

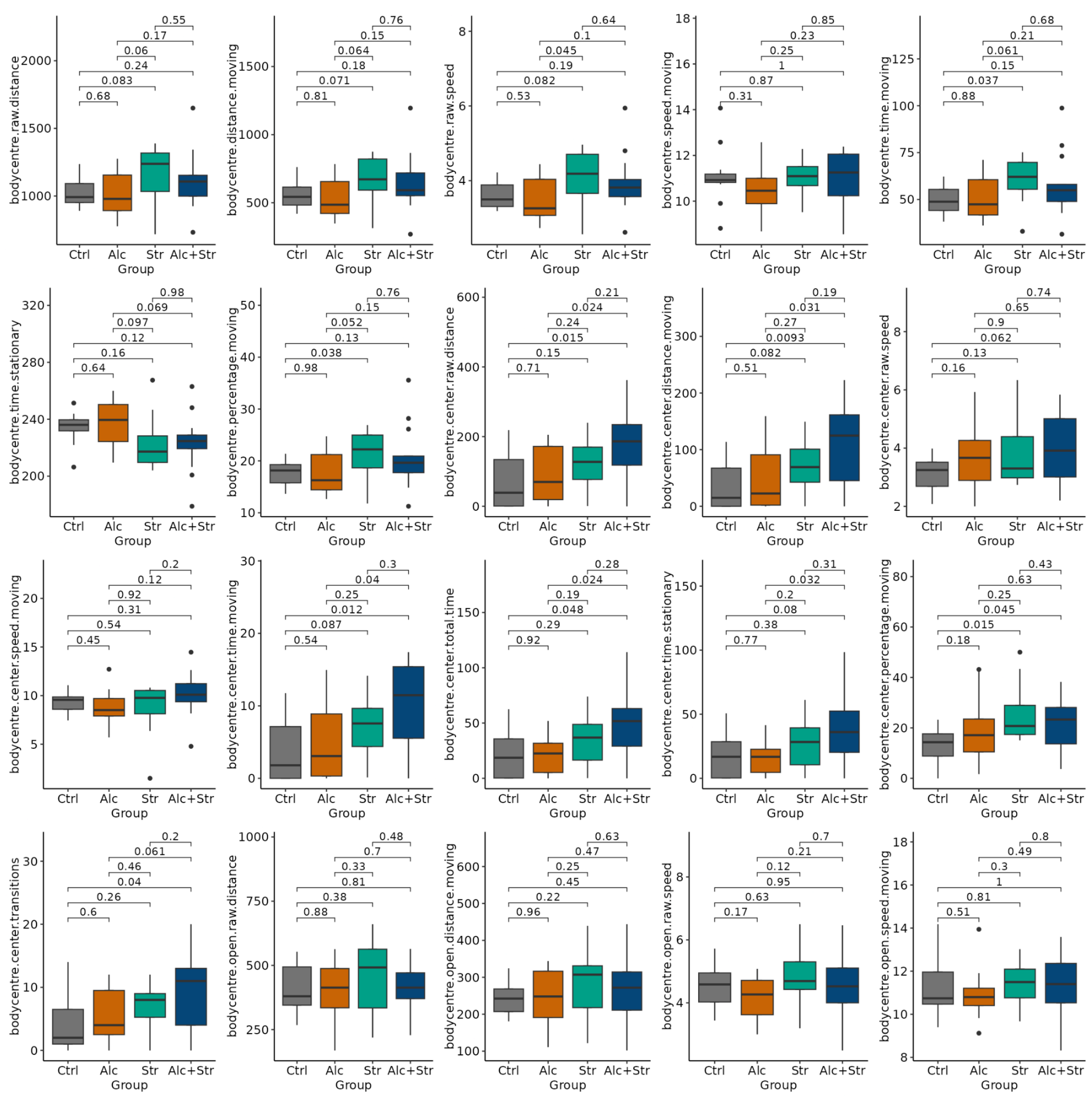

Supplementary figure 11. Individual metrics of elevated plus maze (EPM) and their group differences of the males.

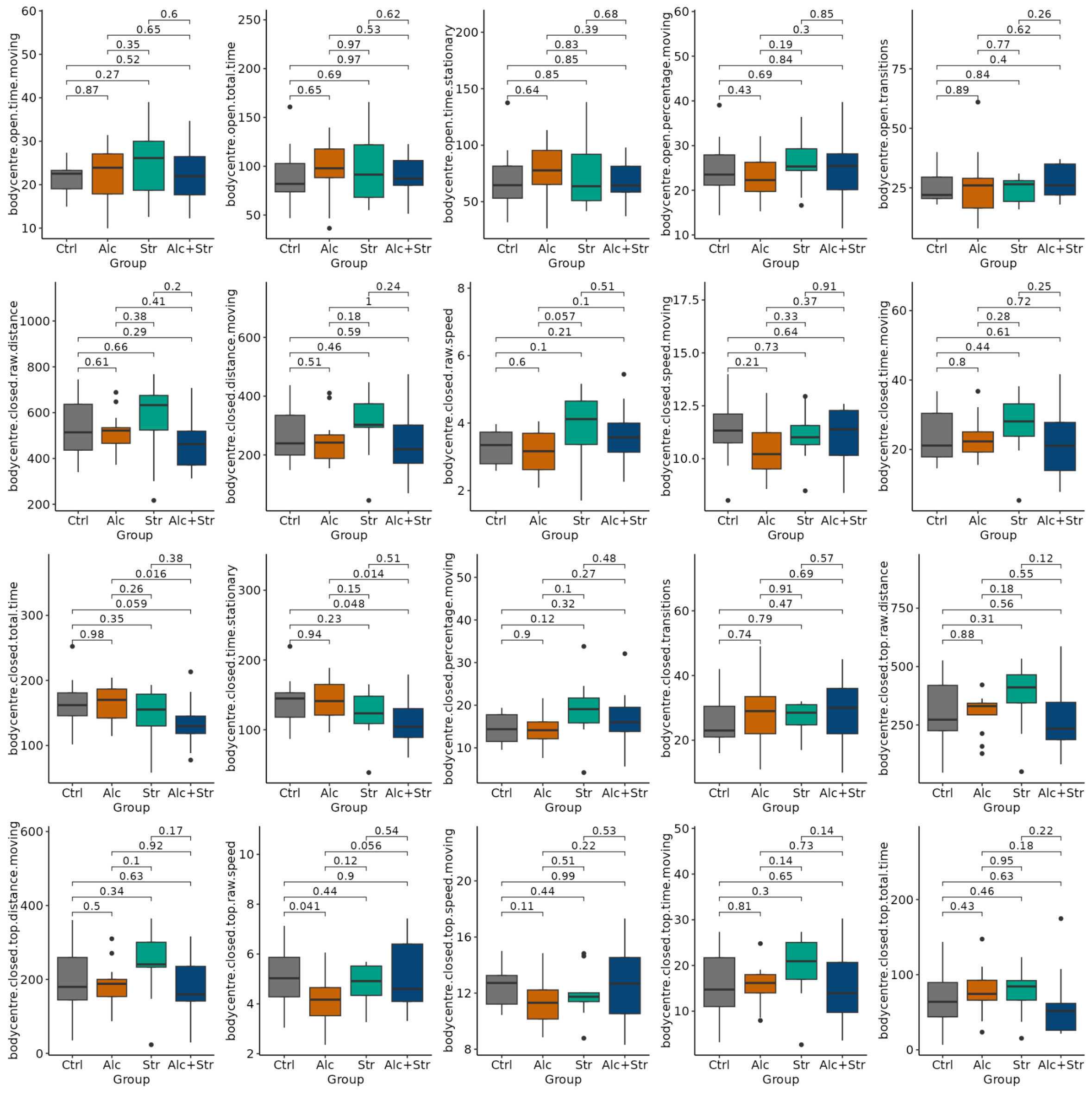

Supplementary figure 11. Individual metrics of elevated plus maze (EPM) and their group differences of the males.

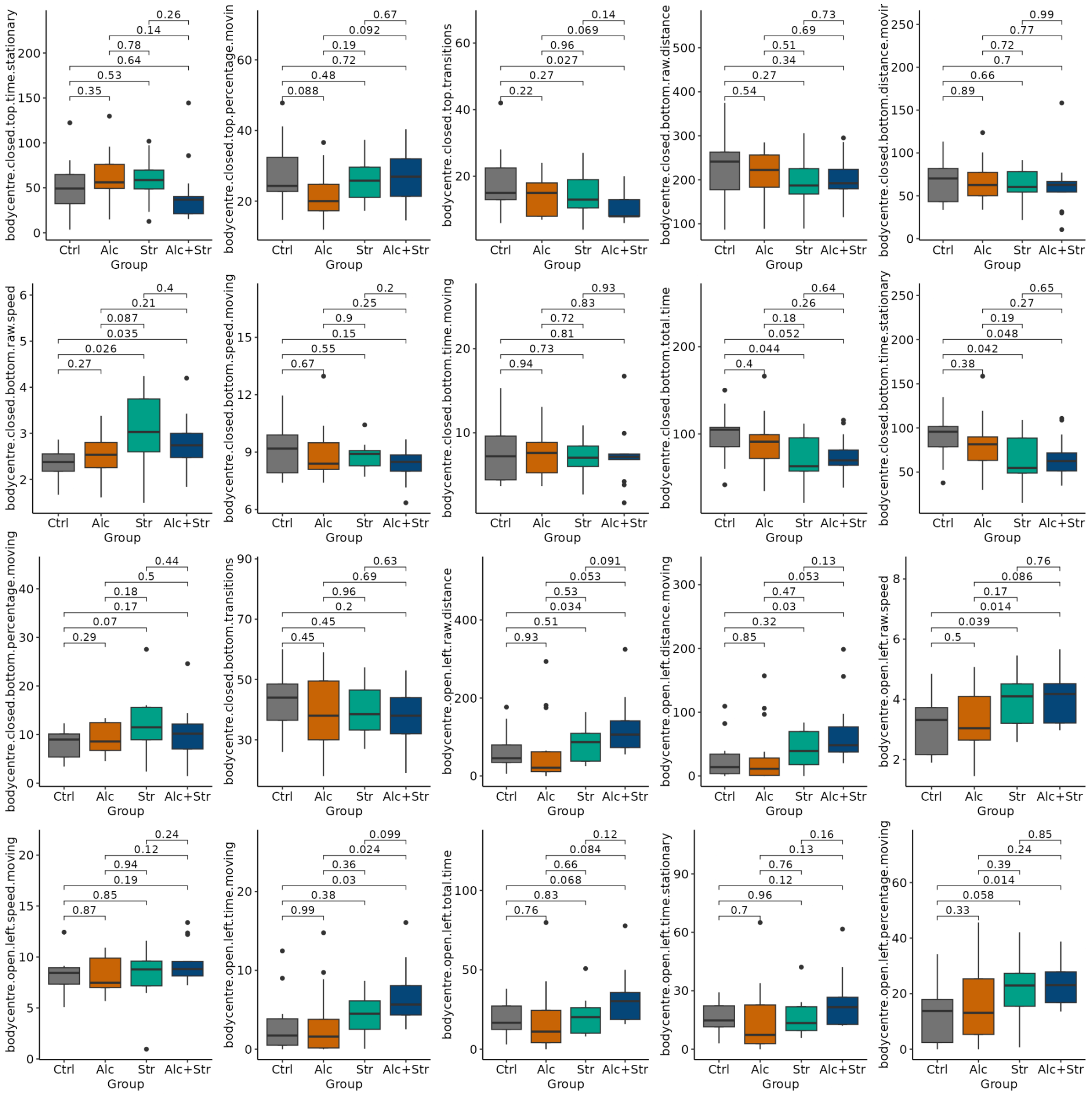

Supplementary figure 11. Individual metrics of elevated plus maze (EPM) and their group differences of the males.

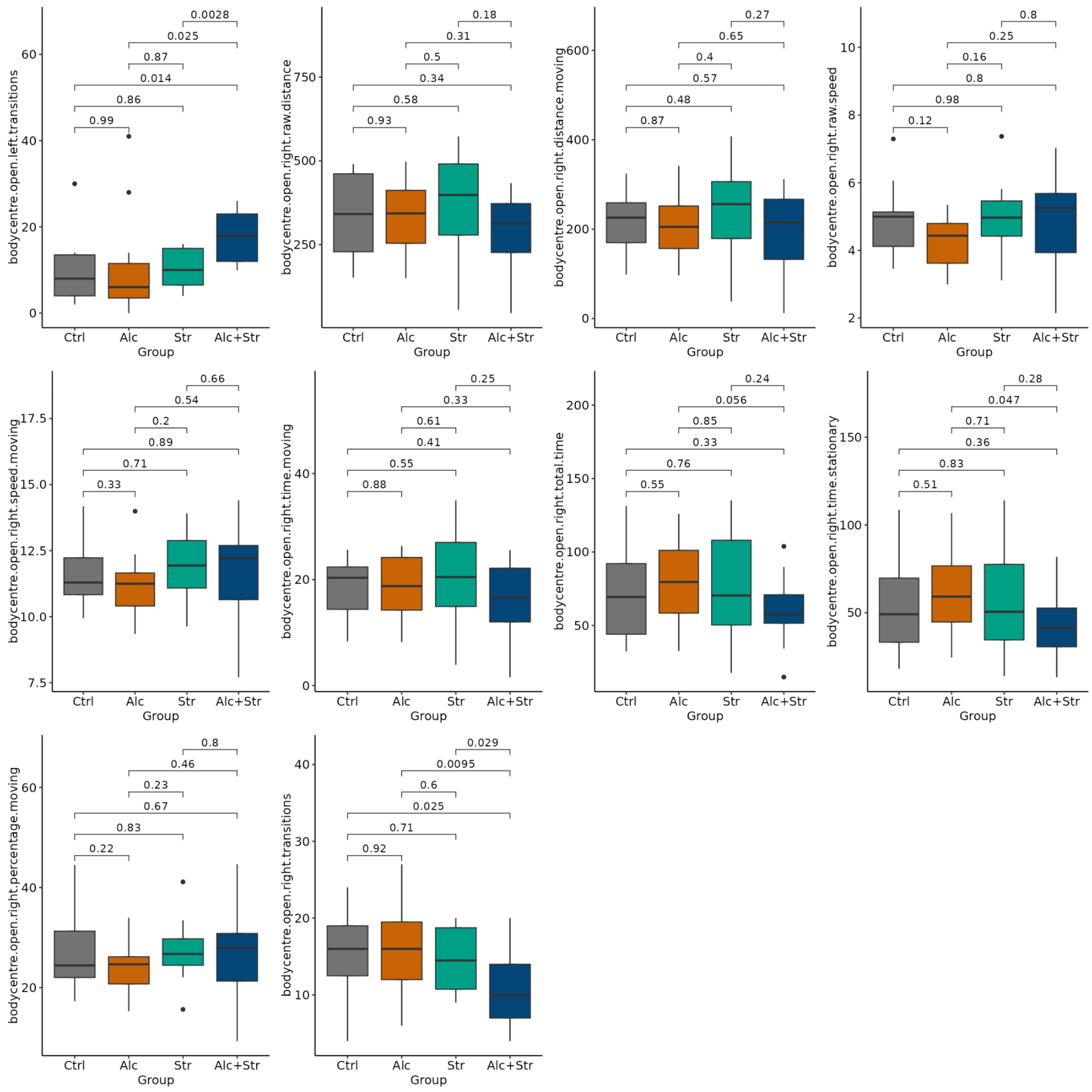

Supplementary figure 12. Individual metrics of novel object recognition (NOR) and their group differences for females

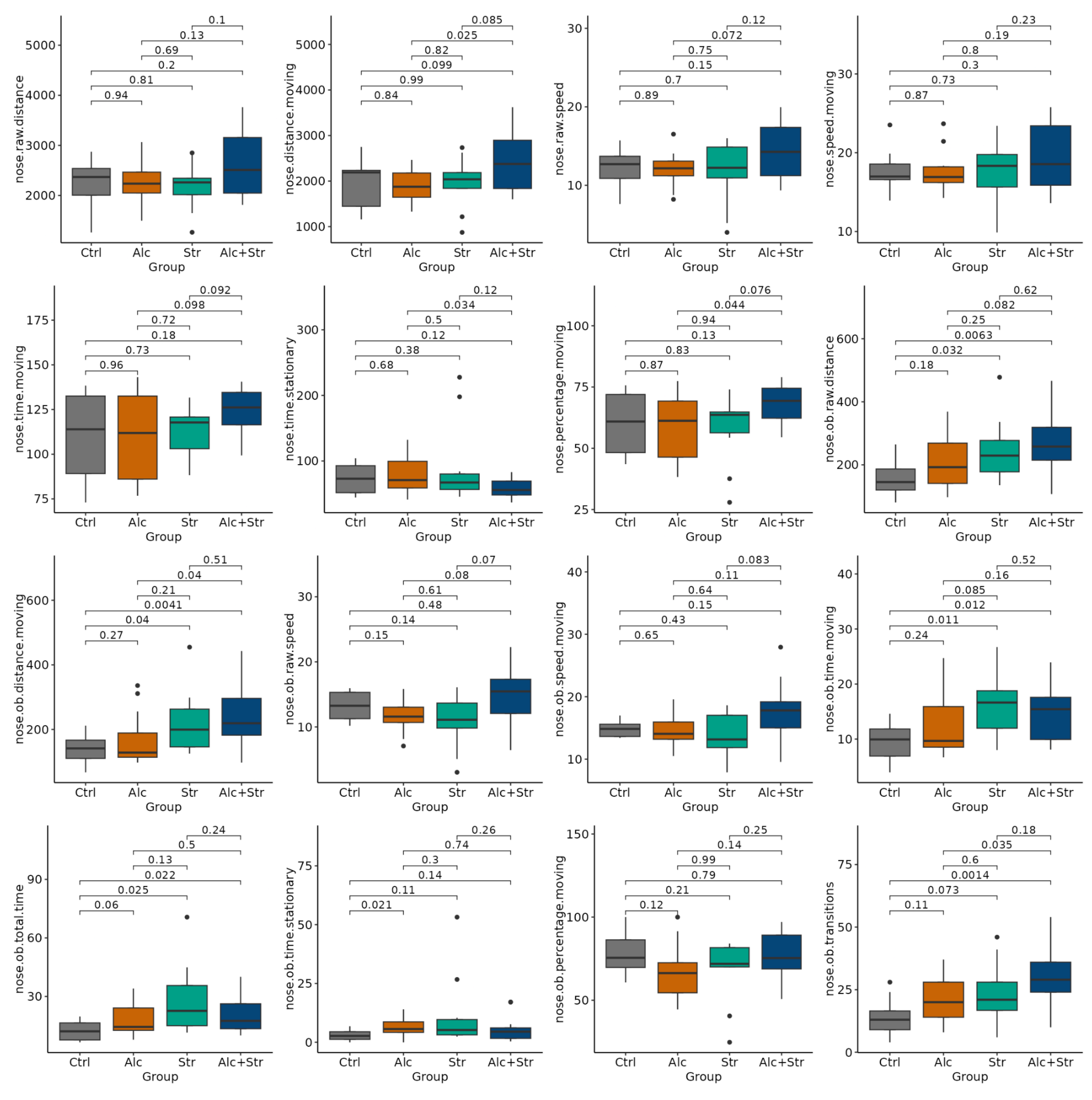

Supplementary figure 12. Individual metrics of novel object recognition (NOR) and their group differences for females

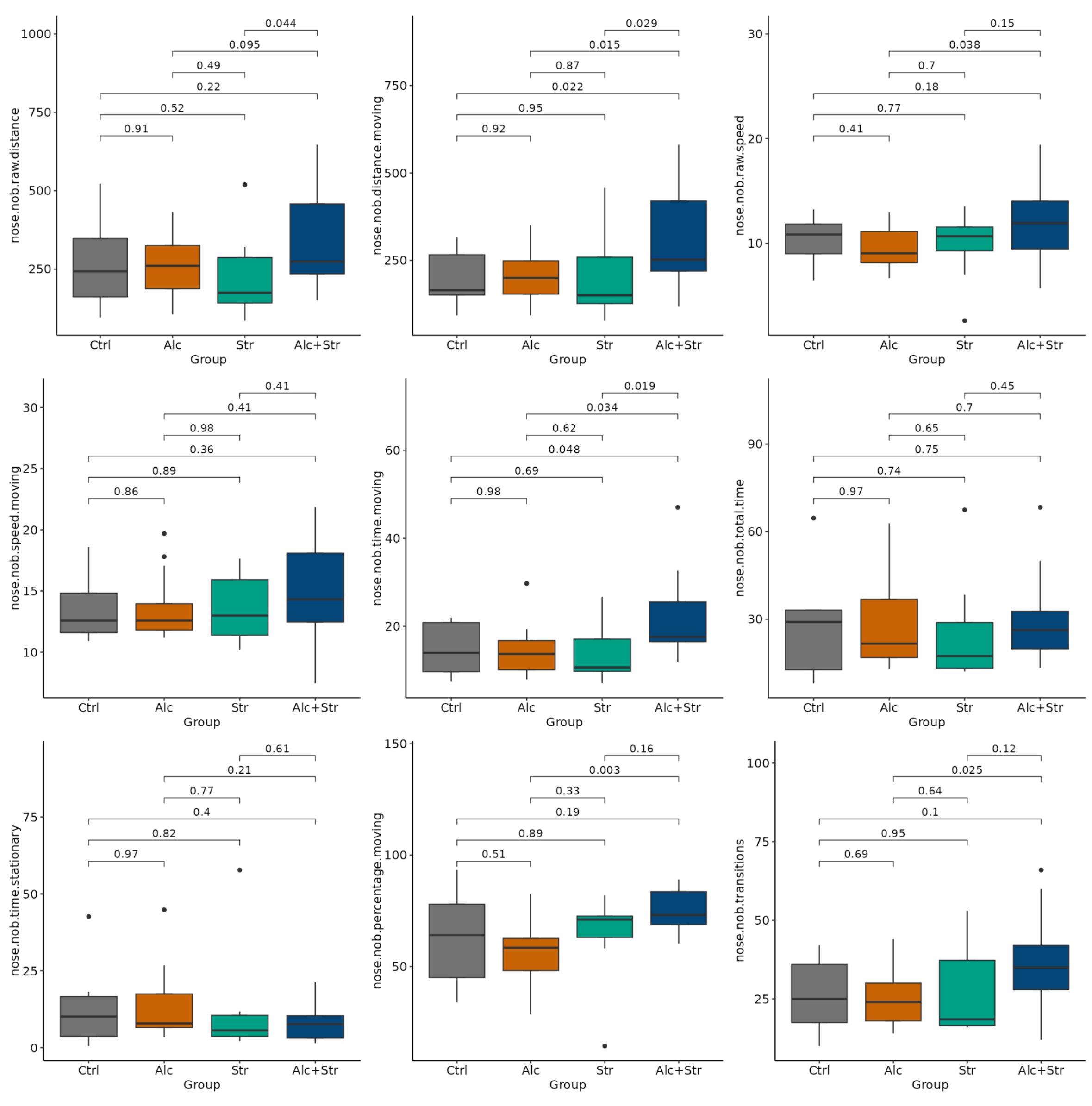

Supplementary figure 12. Individual metrics of novel object recognition (NOR) and their group differences for females

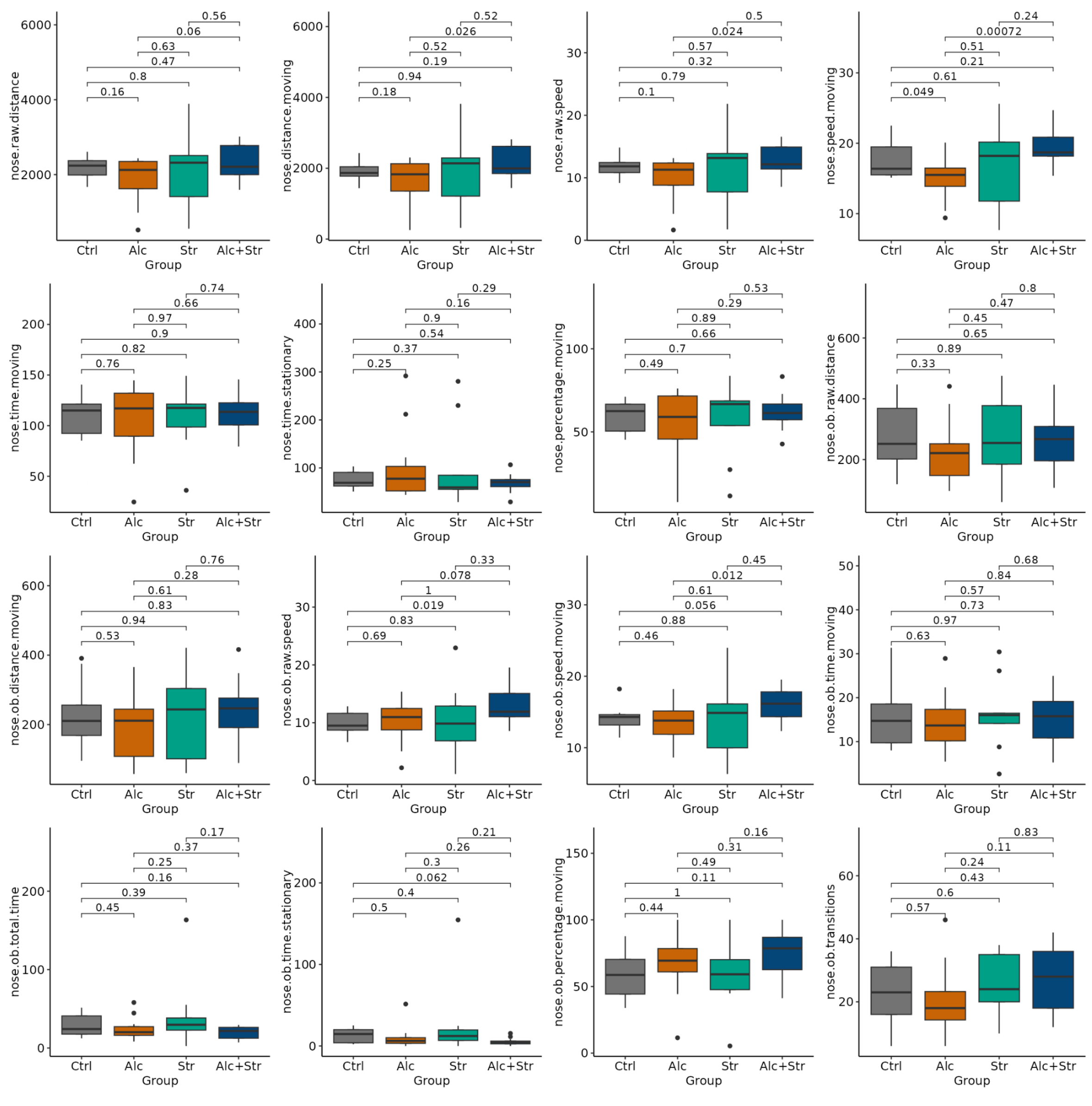

Supplementary figure 12. Individual metrics of novel object recognition (NOR) and their group differences for females

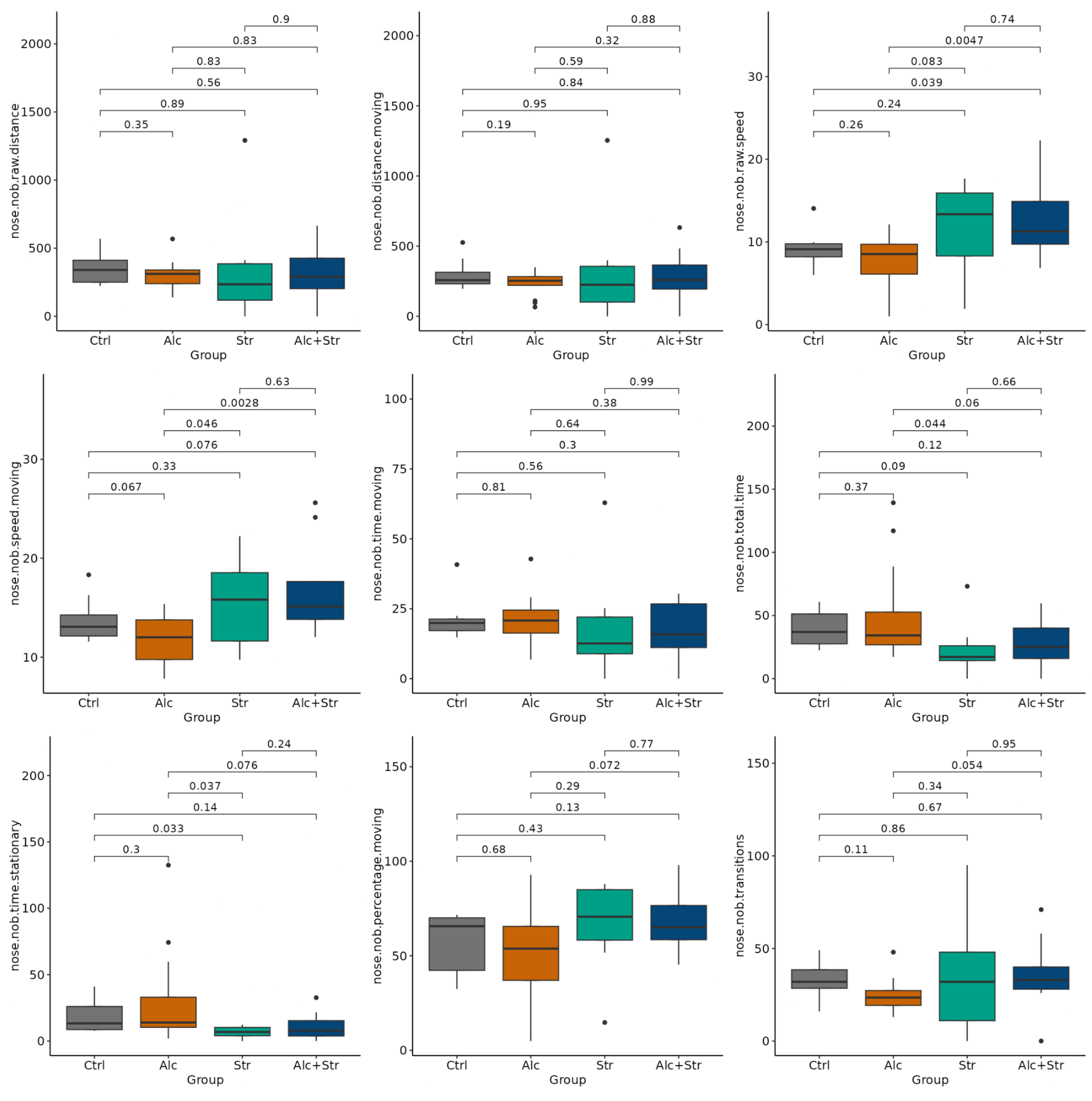

Supplementary discussion

The conditioned place preference (CPP) task revealed that all groups exhibited an equal preference for the ethanol-paired and water-paired compartments. This finding is at odds with the results of studies that reported stress-induced ethanol preference in humans and rodent models. However, it aligns with research demonstrating that age is a significant factor in ethanol preference, as studies have found that adolescent rats do not exhibit a preference for ethanol [1–4].

The findings from the Elevated Plus Maze (EPM) task suggest that ethanol intake and chronic restraint stress (CRS) have differential effects on anxiety-like behaviors in male and female rats. Notably, while the overall anxiety index did not vary significantly among groups, detailed analysis revealed that female rats exposed to ethanol (EtOH+/CRS- and EtOH+/CRS+) exhibited reduced movement and speed, and an increased stationary and total time spent but only in one closed arm. Meanwhile, males exposed to both ethanol and CRS exhibit a more complex pattern with increased exploration in the center and open arms but also increased stationary behavior in the closed arms. These results might imply that ethanol, even after a long withdrawal period, reduces the natural exploratory behavior in females [5,6], while has mixed effects in males depending on their exposure to chronic stress.

Similarly, the results from the Novel Object Recognition (NOR) task showed a reduced discrimination ratio in the male EtOH-/CRS+ group, indicating impaired recognition and memory performance under conditions of chronic stress [7–10]. This observation aligns with previous research demonstrating that chronic stress can adversely affect hippocampal function, leading to memory deficits that vary between sexes [10,11]. Notably, male rats exposed to both ethanol and stress (EtOH+/CRS+) exhibited increased locomotor activity, characterized by greater speed and distance traveled. This behavior may suggest hyperactivity or enhanced exploratory tendencies [12], consistent with studies investigating the effects of chronic stress alone [13] and those examining ethanol exposure independently [14].

Our results further suggest that while the combination of chronic ethanol intake and chronic stress exacerbated the effects on local brain anatomy and functional connectivity network, we also found that each intervention showed distinct patterns of structural and functional neuroadaptations, highly affected by chronic stress more than ethanol intake. In the structural analysis, when we compared the effect of both interventions against the groups with only one intervention (chronic stress or ethanol intake), we found that chronic stress produced an increased volume in Cg2 and RSGc along with a decrease of ventral hippocampus and amygdala, as well as more extensive alterations in local volume within hippocampus, caudate-putamen and amygdala, which are essential neuroanatomical substrates of the stress-response and drug-seeking systems, and intimately involved in processes such as learning, memory, reward and anxiety-like behaviors, which also usually involve more cortical and limbic regions.[15–18] Moreover, we found altered functional connectivity between cortical and subcortical regions (e.g., amygdala-thalamus, amygdala-orbitofrontal cortex, hippocampus-cerebellum, caudate-putamen-orbitofrontal) in the chronic stress group, which is related to both reward systems in addiction and chronic stress. These changes have been previously noted[17], mostly with an FC-decreased pattern, similar to what we observed. However, our findings further reveal that these effects are sex-dependent.[19,20] Therefore, even though chronic stress did not seem to have an effect on ethanol intake, it may be affecting the addiction processes by either influencing dependency or relapse. This could give us an insight into the addictive effect of pathological chronic stress on morphology, functionality, and memory that might be caused by microstructural processes.[21,22].

References

1. Song M, Wang XY, Zhao M, Wang XY, Zhai HF, Lu L. Role of stress in acquisition of alcohol-conditioned place preference in adolescent and adult mice. *Alcohol Clin Exp Res*. 2007;31(12):2001-2005. doi:10.1111/j.1530-0277.2007.00522.x

2. Kuhns L, Kroon E, Lesscher H, Mies G, Cousijn J. Age-related differences in the effect of chronic alcohol on cognition and the brain: a systematic review. *Transl Psychiatry*. 2022;12(1):345. doi:10.1038/s41398-022-02100-y

3. Yu L, Wang L, Zhao X, Song M, Wang X. Role of single prolonged stress in acquisition of alcohol conditioned place preference in rats. *Life Sci*. 2016;151:259-263. doi:10.1016/j.lfs.2016.03.004

4. Wille-Bille A, Ferreyra A, Sciangula M, Chiner F, Nizhnikov ME, Pautassi RM. Restraint stress enhances alcohol intake in adolescent female rats but reduces alcohol intake in adolescent male and adult female rats. *Behav Brain Res*. 2017;332:269-279. doi:10.1016/j.bbr.2017.06.004

5. Mittal N, Fleming SM, Martinez A, et al. Sex differences in cognitive performance and alcohol consumption in High Alcohol-Drinking (HAD-1) rats. *Behav Brain Res*. 2020;381:112456. doi:10.1016/j.bbr.2019.112456

6. Knight P, Chellian R, Wilson R, Behnood-Rod A, Panunzio S, Bruijnzeel AW. Sex differences in the elevated plus-maze test and large open field test in adult Wistar rats. *Pharmacol Biochem Behav*. 2021;204:173168. doi:10.1016/j.pbb.2021.173168

7. Chanraud S, Pitel AL, Rohlfing T, Pfefferbaum A, Sullivan EV. Dual tasking and working memory in alcoholism: relation to frontocerebellar circuitry. *Neuropsychopharmacology*. 2010;35(9):1868-1878. doi:10.1038/npp.2010.56

8. de Quervain DJ, Roozendaal B, McGaugh JL. Stress and glucocorticoids impair retrieval of long-term spatial memory. *Nature*. 1998;394(6695):787-790. doi:10.1038/29542

9. Warburton EC, Brown MW. Neural circuitry for rat recognition memory. *Behav Brain Res*. 2015;285:131-139. doi:10.1016/j.bbr.2014.09.050

10. Woo H, Hong CJ, Jung S, Choe S, Yu SW. Chronic restraint stress induces hippocampal memory deficits by impairing insulin signaling. *Mol Brain*. 2018;11(1):37. doi:10.1186/s13041-018-0381-8

11. Bowman RE, Beck KD, Luine VN. Chronic stress effects on memory: sex differences in performance and monoaminergic activity. *Horm Behav*. 2003;43(1):48-59. doi:10.1016/s0018-506x(02)00022-3

12. Knardahl S, Sagvolden T. Open-field behavior of spontaneously hypertensive rats. *Behav Neural Biol*. 1979;27(2):187-200. doi:10.1016/s0163-1047(79)91801-6

13. Marin MT, Cruz FC, Planeta CS. Chronic restraint or variable stresses differently affect the behavior, corticosterone secretion and body weight in rats. *Physiol Behav*. 2007;90(1):29-35. doi:10.1016/j.physbeh.2006.08.021

14. Cacace S, Plescia F, La Barbera M, Cannizzaro C. Evaluation of chronic alcohol self-administration by a 3-bottle choice paradigm in adult male rats. Effects on behavioural reactivity, spatial learning and reference memory. *Behav Brain Res*. 2011;219(2):213-220. doi:10.1016/j.bbr.2011.01.004

15. Gozzi A, Agosta F, Massi M, Ciccocioppo R, Bifone A. Reduced limbic metabolism and fronto-cortical volume in rats vulnerable to alcohol addiction. *Neuroimage*. 2013;69:112-119. doi:10.1016/j.neuroimage.2012.12.015

16. Coleman LG Jr, Liu W, Oguz I, Styner M, Crews FT. Adolescent binge ethanol treatment alters adult brain regional volumes, cortical extracellular matrix protein and behavioral flexibility. *Pharmacol Biochem Behav*. 2014;116:142-151. doi:10.1016/j.pbb.2013.11.021

17. Magalhães R, Barrière DA, Novais A, et al. The dynamics of stress: a longitudinal MRI study of rat brain structure and connectome. *Mol Psychiatry*. 2018;23(10):1998-2006. doi:10.1038/mp.2017.244

18. Gaser C, Nenadic I, Buchsbaum BR, Hazlett EA, Buchsbaum MS. Deformation-based morphometry and its relation to conventional volumetry of brain lateral ventricles in MRI. *Neuroimage*. 2001;13(6 Pt 1):1140-1145. doi:10.1006/nimg.2001.0771

19. Arnsten AFT. Stress signalling pathways that impair prefrontal cortex structure and function. *Nat Rev Neurosci*. 2009;10(6):410-422. doi:10.1038/nrn2648

20. Luine VN, Beck KD, Bowman RE, Frankfurt M, Maclusky NJ. Chronic stress and neural function: accounting for sex and age. *J Neuroendocrinol*. 2007;19(10):743-751. doi:10.1111/j.1365-2826.2007.01594.x

21. Atrooz F, Alkadhi KA, Salim S. Understanding stress: Insights from rodent models. *Curr Res Neurobiol*. 2021;2:100013. doi:10.1016/j.crneur.2021.100013

22. Kim EJ, Kim JJ. Neurocognitive effects of stress: a metaparadigm perspective. *Mol Psychiatry*. 2023;28(7):2750-2763. doi:10.1038/s41380-023-01986-4
